# Beyond the polymerase-γ theory: Production of ROS as a mode of NRTI-induced mitochondrial toxicity

**DOI:** 10.1371/journal.pone.0187424

**Published:** 2017-11-02

**Authors:** Reuben L. Smith, Josephine M. E. Tan, Martijs J. Jonker, Aldo Jongejan, Thomas Buissink, Steve Veldhuijzen, Antoine H. C. van Kampen, Stanley Brul, Hans van der Spek

**Affiliations:** 1 Molecular Biology & Microbial Food Safety, Swammerdam Institute for Life Sciences (SILS), Faculty of Science (FNWI), University of Amsterdam, Amsterdam, The Netherlands; 2 RNA Biology & Applied Bioinformatics, Swammerdam Institute for Life Sciences (SILS), Faculty of Science (FNWI), University of Amsterdam, Amsterdam, The Netherlands; 3 Bioinformatics Laboratory, Clinical Epidemiology, Biostatistics and Bioinformatics, Academic Medical Center (AMC), Amsterdam, The Netherlands; 4 Biosystems data analysis, Swammerdam Institute for Life Sciences (SILS), Faculty of Science (FNWI), University of Amsterdam, Amsterdam, The Netherlands; University of Nebraska-Lincoln, UNITED STATES

## Abstract

Use of some HIV-1 nucleoside reverse transcriptase inhibitors (NRTI) is associated with severe adverse events. However, the exact mechanisms behind their toxicity has not been fully understood. Mitochondrial dysfunction after chronic exposure to specific NRTIs has predominantly been assigned to mitochondrial polymerase-γ inhibition by NRTIs. However, an increasing amount of data suggests that this is not the sole mechanism. Many NRTI induced adverse events have been linked to the incurrence of oxidative stress, although the causality of events leading to reactive oxygen species (ROS) production and their role in toxicity is unclear. In this study we show that short-term effects of first generation NRTIs, which are rarely discussed in the literature, include inhibition of oxygen consumption, decreased ATP levels and increased ROS production. Collectively these events affect fitness and longevity of *C*. *elegans* through mitohormetic signalling events. Furthermore, we demonstrate that these effects can be normalized by addition of the anti-oxidant *N*-acetylcysteine (NAC), which suggests that ROS likely influence the onset and severity of adverse events upon drug exposure.

## Introduction

Modern antiretroviral medication has improved considerably since the introduction of 3’-azido-3’-deoxythymidine (*zidovudine* or AZT) in 1987 and is considered one of the major medical advances of modern medicine due to its efficacy in suppressing HIV-1 replication and maternal transmission. Nonetheless, antiretroviral medicines are frequently associated with severe adverse events and the initiation or exacerbation of degenerative processes, diseases and syndromes [1]. Mitochondrial toxicity caused specifically by NRTI induced perturbation of the function of mitochondrial DNA (mtDNA) polymerase-γ has been denoted as a central mechanism underlying these adverse events and is commonly referred to as ‘the polymerase-γ theory’. In short, polymerase-γ is responsible for mtDNA replication and repair. Inhibition of this enzyme would then result in reduced mtDNA integrity and copy number [2]. As mtDNA encodes for essential components of the mitochondrial respiratory chain, depletion in mtDNA quality and quantity impedes mitochondrial oxidative phosphorylation and consequently mitochondrial function. Lower ATP levels, diminished mitochondrial membrane potential and elevated ROS have frequently been shown to result from exposure to NRTIs *in vitro* [3].

### Toxicity beyond the polymerase-γ theory

Although the polymerase-γ theory clarified several of the adverse events witnessed in patients receiving NRTIs, a large body of evidence has accumulated over the years which suggests that there are modes to NRTI induced mitochondrial toxicity that lie beyond inhibition of polymerase-γ [4,5]. Affinity with and concurrent inhibition of polymerase-γ by NRTIs does not linearly correlate with clinical manifestations of mitochondrial toxicity. Of the NRTIs, AZT for example does not have the highest potential to inhibit polymerase-γ, yet it is the NRTI that is associated with the most mitochondrial related adverse events [6]. Additionally, mitochondrial toxicity caused by NRTIs does not necessarily follow the chronological steps of the polymerase-γ theory; not every case of mtDNA depletion leads to changed expression or activity of mitochondrial respiratory chain (MRC) proteins [7,8]. On the other hand, altered mitochondrial gene transcription and impaired respiratory chain activity have been observed in the absence of mtDNA depletion or reduction of polypeptide synthesis [9–11].

In light of this, NRTIs have been proposed to interfere with mitochondrial function through other mechanisms like altered nucleoside homeostasis caused by NRTI pharmacokinetics [12]. NRTIs can also directly inhibit mitochondrial enzymes [13] and are known to enhance the generation of ROS [14,15] (for a comprehensive review see [4]).

### Reactive oxygen species

Of the models for NRTI toxicity beyond the polymerase-γ theory, oxidative stress is considered to be the most conspicuous [14]. This is especially the case when taking into account that polymerase-γ is sensitive to oxidative damage and modification of its amino acid residues by oxidation causes a decrease in DNA-binding ability and polymerase activity [16]. Many NRTI induced detrimental processes have been linked to the incurrence of oxidative stress [14,15]. For example, cardio-vascular disease, central nervous system disorders, inflammation, and metabolic and lipodystrophy syndromes have all been found to be related to ROS caused by antiretroviral treatment [17–19]. While the damaging capacities of ROS are indisputable, more recent data shows that ROS are also important signalling molecules that regulate fundamental cellular processes such as apoptosis, mitophagy and autophagy, immune responses, and adaptation to hypoxia, starvation and stress [20–22]. HIV-1 patients treated with antiretroviral therapy have been shown to have significantly higher serum oxidant levels compared to therapy naive HIV-1 patients and uninfected controls. Underscoring the effect of antiretroviral therapy on oxidative stress, patients who strictly adhere to therapy guidelines have increased oxidant levels and lower antioxidant levels compared to those who do not closely follow therapy [23]. Collectively, these observations indicate that oxidative stress may be a powerful driving force behind antiretroviral induced toxicity and may have prominent roles in the onset and exacerbation of many detrimental conditions [1,14].

### Time-dependent effects of NRTIs

The initiation of mitochondrial dysfunction by NRTIs has predominantly been studied after chronic drug exposure, wherein the chronology of events as proposed in the polymerase-γ theory have time to take place and many adverse events can consequently develop. The short-term effects of these drugs, however, have not been subject of study. It is essential to recognize the initial effects NRTIs have on mitochondria if we are to understand their long-term detrimental effects. Short term mitochondrial dysfunction and ROS signalling are known to prompt the mitochondria into activating various stress responses which result in long term changes in mitochondrial function [20,24]. We therefore set out to investigate the role of ROS in NRTI induced adverse events using *Caenorhabditis elegans* as a model system using HIV-1 drugs that are known to cause mitochondrial toxicity in patients. Our results suggest that NRTIs increase ROS production likely through direct inhibition the mitochondrial respiratory chain (MRC) and that these events affect fitness and longevity of *C*. *elegans* through mitohormetic signalling events.

## Results

### ROS production may underlie NRTI induced variations in mtDNA copy number

**Fig 1:**
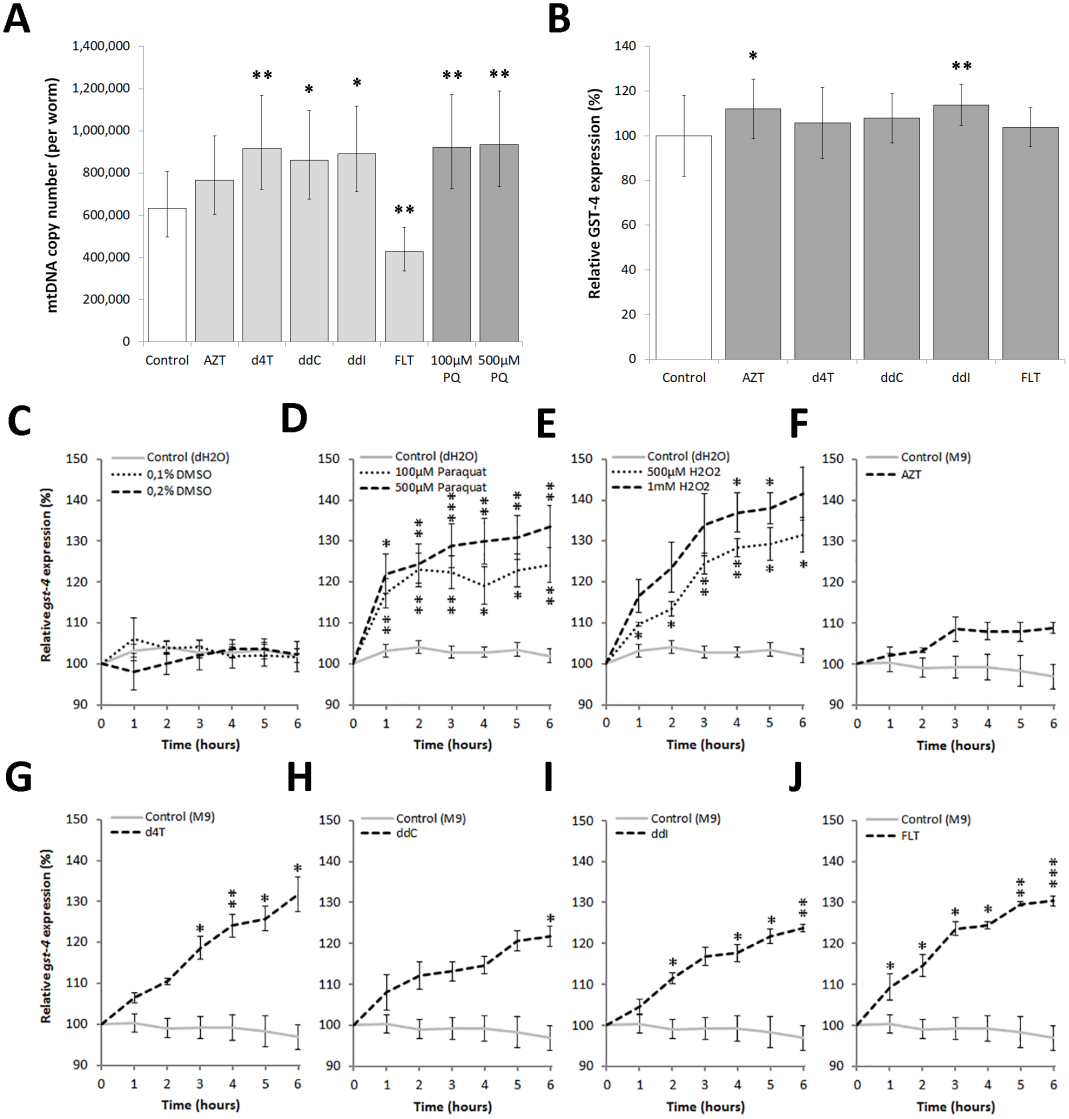
**A: Chronic NRTI exposure induced changes in mtDNA copy number**. A: Compared to control nematodes, after 72h exposure, FLT is the only NRTI that reduced mtDNA copy number. d4T, ddC & ddI enhanced mtDNA copy numbers, whereas AZT showed no significant change. 100μM and 500μM paraquat also increased mtDNA copy number. Error bars show the 95% C.I. (df = 51) of ≥6 biological replicates from 2 individual experiments. **B: Chronic NRTI exposure influences GST-4 expression**: Relative *gst-4* expression levels were higher for AZT and ddI after 24h exposure. Significance was determined using a one way ANOVA and Turkey’s multiple comparisons test, compared to control animals (≥5 replicates per 2 individual experiments). NRTI concentration = 200μM. **C-J: NRTIs increase the expression of *gst-4* similar to the pro-oxidants paraquat and hydrogen peroxide**. C: Relevant DMSO concentrations (4 replicates per 5 individual experiments); D: paraquat (4 replicates per 5 individual experiments); E: H_2_O_2_ (5 replicates per 4 individual experiments); F: AZT; G: d4T; H: ddC; I: ddI; and J: FLT. Results depict continuous exposure of therapy naïve animals to 200μM NRTIs during 6 hours (≥4 replicates per 3 individual experiments). Pro-oxidant and DMSO analyses were calculated using control dH_2_O (≥3 replicates per 13 individual experiments) and NRTI analyses were calculated using the relevant DMSO concentration as controls (AZT, d4T, ddI, FLT = 0.1% DMSO; ddC = 0.2% DMSO). Significance was calculated using a multifactorial ANOVA without replication compared to relevant controls. * = P<0.05, ** = P<0.01, *** = P<0,001.

Previously we demonstrated that nematodes chronically exposed to selective NRTIs have perturbed mitochondrial morphology and decreased respiration, corroborating the notion that NRTIs are toxic to mitochondria [25]. To assess if chronic exposure to NRTIs reduced mtDNA copy number as proposed by the polymerase-γ theory, we performed a quantitative PCR after 72h exposure of L4 animals to 200μM NRTIs (Fig 1A). MtDNA copy number only decreased with 3’-deoxy-3’-fluorothymidine (a*lovudine* or FLT), whereas 2’,3’-didehydro-2’,3’-deoxythymidine (*stavudine* or d4T), 2’,3’-dideoxycytidine (*zalcitabine* or ddC) and 2’,3’-dideoxyinosine (*didanosine* or ddI) showed elevated copy numbers. AZT showed no significant change. Interestingly, 100μM and 500μM of the superoxide generating agent paraquat also caused mtDNA copy numbers to increase (Fig 1A). These results clearly cannot be solely explained by polymerase-γ inhibition by the NRTIs or by confounding effects of germ cell production—which involves a very high level of mtDNA replication—as these nematodes were treated with FuDR. Moreover, changes in mtDNA copy number upon paraquat exposure indicates that ROS can influence mtDNA copy number.

### NRTIs rapidly induce ROS defence mechanisms

The nematode specific glutathione *S*-transferase 4 (GST-4) has been shown to be directly involved in resistance to oxidative stress [26]. After 24h exposure to NRTIs, only AZT and ddI showed slight yet significant upregulation of *gst-4* expression (Fig 1B). NRTIs have been shown to induce peak levels of mitochondrially derived ROS in human umbilical vein endothelial cells within 8h exposure, which returns to levels similar to the control after 24h [27]. We therefore surmised that any burst in mitochondrial ROS production caused by NRTIs must take place earlier than 24h. During six hours of exposure, NRTIs significantly increased *gst-4* expression (Fig 1F–1J). *Gst-4* expression was also rapidly induced upon exposure to hydrogen peroxide (H_2_O_2_) and paraquat in a dose dependent manner (Fig 1D & 1E). The NRTI solvent DMSO showed no significant change in *gst-4* expression over time at relevant concentrations (Fig 1C).

### Short term NRTI and pro-oxidant exposure leads to changes in mtDNA copy number

**Fig 2:**
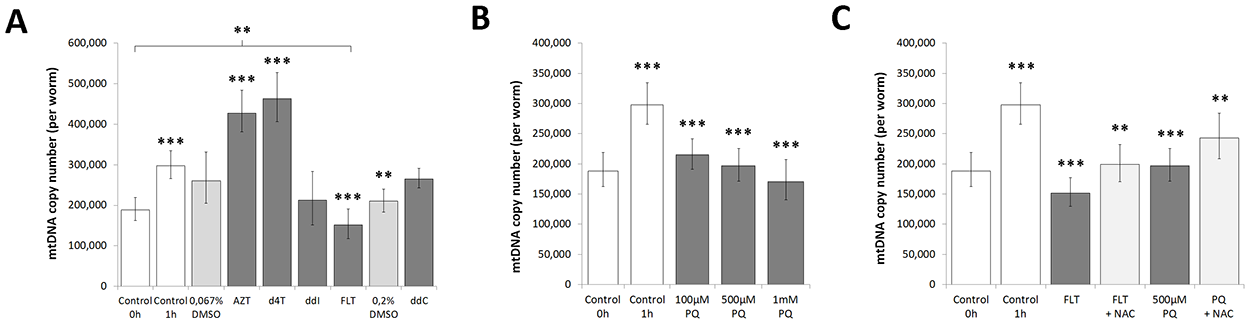
**A: mtDNA copy number was altered upon short term (1h) exposure to NRTIs** and **B: paraquat**. A: 200μM NRTIs and B: paraquat induced changes in mtDNA copy number compared to their respective controls after 1h exposure: 0.067% DMSO for AZT, d4T, ddI & FLT; 0.2% DMSO for ddC; and control for paraquat. mtDNA copy number increases during nematode development and therefore rose after 1h (control 0h vs control 1h). FLT & paraquat inhibited normal mtDNA copy number increase. AZT & d4T exposure resulted in higher mtDNA copy numbers. 0.2% DMSO reduced mtDNA copy number. Error bars show the 95% C.I. (51df) from ≥3 replicates per 2 individual experiments. NRTIs and paraquat were compared to control animals at 1h. **C: mtDNA copy number decrease by FLT and paraquat was attenuated by NAC**. 200μM FLT and 500μM paraquat decreased mtDNA copy number compared to control nematodes after 1h exposure (dark grey vs 1h control). Supplementation of an anti-oxidant (NAC) attenuated this decline (light grey vs dark grey). Anti-oxidant concentration = 100μM. Error bars show the 95% C.I. (51df) from ≥3 replicates per 2 individual experiments. Control animals at 1h compared to FLT and paraquat, and drugs + anti-oxidants compared to drugs only, and Control 1h vs Control 0h. Significance was determined using a two-tailed student’s *t*-test assuming unequal variance. * = P-value <0.05, ** = P-value <0.01, *** = P-value <0.001.

MtDNA replication in *C*. *elegans* predominantly occurs in the proliferating gonads at late larval and early adult stages [28]. Specifically, mtDNA copy number rises sharply from approximately 250.000 in the L4 stage to 600.000 in D1 adults [29]. We used this phenomenon to our advantage and by exposing L4 nematodes to antiretrovirals for a period of 6 hours found that they can rapidly induce fluctuations in mtDNA copy number (Fig 2A & Table 1) without observing any effects on growth. As expected, we detected that during nematode development from the larval L4 stage into young-adulthood, the mtDNA number increased sharply from approximately 200.000 copies to 300.000 within 1h. In comparison to their respective 1h DMSO control, AZT and d4T amplified this increase, whereas FLT decreased mtDNA copy numbers below even that of control animals at 0h. ddC and ddI showed no effect (Fig 2A). Interestingly, paraquat showed a dose dependent decline in mtDNA copy number compared to control animals at 1h (Fig 2B). Continued exposure to AZT, d4T, ddC, ddI, and paraquat, showed that over time mtDNA copy numbers first decline and then gradually approach values comparable to the control. MtDNA copy numbers for FLT, however, remained suppressed (Table 1), reaffirming the strong inhibitory role of FLT on polymerase-γ [30].

**Table 1:** mtDNA copy numbers fluctuated and eventually became normalized during continued short-term exposure (6h). Relative quantities (%) of mtDNA compared to control animals. Numbers between parentheses indicate 95% confidence intervals (51df) from ≥3 replicates per 2 individual experiments. Significance was determined using a two-tailed student’s *t*-test assuming unequal variance compared to control animals. * = P-value <0.05, ** = P-value <0.01, *** = P-value <0.001.

	2h (-/+)	3h (-/+)	4h (-/+)	5h (-/+)	6h (-/+)
**Control**	100 (21/27)	100 (21/27)	100 (21/27)	100 (21/27)	100 (21/27)
**AZT**	78 (17/21)	76 (16/21)	76 (16/21) *	106 (23/29)	97 (24/33)
**d4T**	81 (17/22)	73 (16/20) *	75 (16/20) *	73 (18/25)	107 (22/27)
**ddC**	79 (17/22)	68 (15/19) **	74 (14/17) *	131 (28/36)	132 (33/44)
**ddI**	76 (16/21) *	76 (16/21) *	82 (18/23)	83 (21/28)	111 (24/30)
**FLT**	25 (16/20) *	55 (12/15) ***	70 (15/19) *	94 (20/26)	59 (13/16) ***
**100μM PQ**	85 (18/23)	73 (16/20) *	94 (20/26)	134 (29/36) *	112 (28/38)
**500μM PQ**	90 (19/24)	84 (18/23)	85 (18/23)	103 (22/28)	106 (19/24)
**1mM PQ**	82 (18/22)	72 (15/20) *	98 (18/22)	129 (26/32)	100 (25/34)

### Antioxidants attenuate short-term FLT induced mtDNA copy number decline

As it is unclear how the NRTIs reduce mtDNA replication within such a short time frame, we investigated the role of ROS in NRTI induced mtDNA copy number decrease by testing the ability of 100μM *N*-acetylcysteine (NAC) to attenuate the immediate decline of mtDNA copy number caused by FLT. Supplementation with NAC significantly attenuated the FLT induced reduction in mtDNA copy number. Paraquat-induced decline in mtDNA copy number was also attenuated by NAC (Fig 2C). 100μM NAC alone caused a small yet significant decrease in mtDNA replication after 1h exposure (S1 Fig). Taken together, these results suggest that the regulation of mtDNA replication can be significantly altered by cellular redox state and that the rapid decline in mtDNA copy numbers observed upon exposure to FLT is, in part, dependent on an increase in ROS production.

### MRC function is immediately perturbed upon exposure to NRTIs

**Fig 3:**
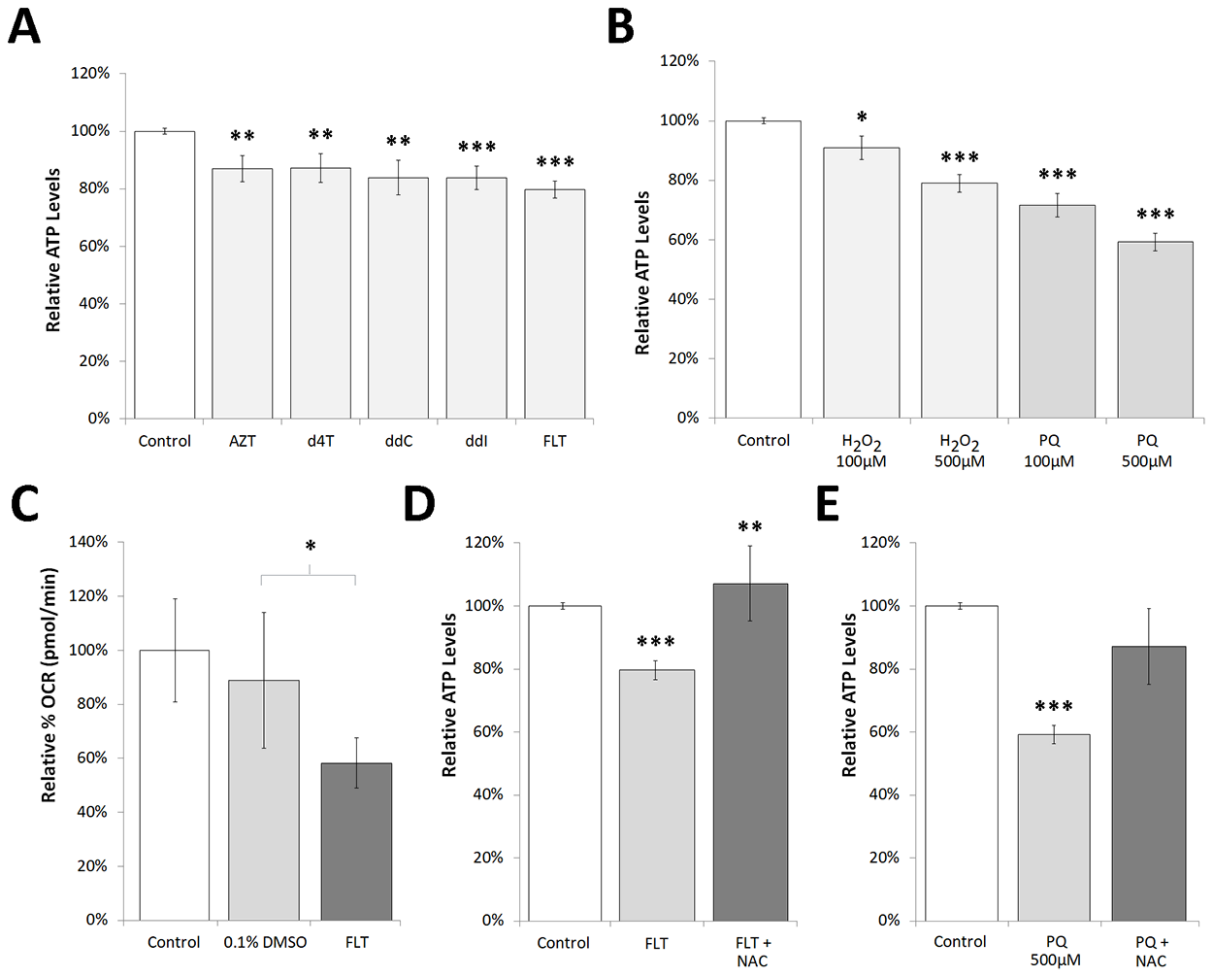
**A: ATP levels were immediately perturbed upon exposure to NRTIs** and **B: pro-oxidants paraquat (PQ) and hydrogen peroxide (H**_**2**_**O**_**2**_**)**. Relative ATP levels (%) declined significantly within 2.5 minutes upon exposure to NRTIs (≥7 replicates in 4 individual experiments) and pro-oxidants (≥7 replicates in 3 individual experiments) *in vivo*, compared to the control (0.33% DMSO). **C: OCR was immediately reduced upon exposure to FLT**. Oxygen consumption rate (OCR) was reduced within 5 minutes exposure to FLT. Statistics were calculated with a one way ANOVA with Dunnett’s multiple comparisons test. Error bars indicate standard error for ≥2 replicates of 20 worms per ≥3 conditions. **D: ATP level decrease by FLT** and **E: paraquat were attenuated upon exposure to 100μM NAC**. ATP levels were measured at 2.5 minutes after exposure *in vivo*. ATP statistics were calculated with a two-way ANOVA with replication. FLT and PQ compared to control and FLT and PQ + anti-oxidant compared to FLT or PQ alone (≥3 replicates in 3 individual experiments). * = P<0.05, ** = P<0.01, *** = P<0.001. NRTI concentration = 200μM.

NRTIs, in particular AZT, have been suggested to directly inhibit the mitochondrial respiratory chain complex I (NADH ubiquinone oxidoreductase), complex II (succinate dehydrogenase), and complex V (F_1_,F_0_-ATPase) [31–33]. We therefore measured MRC function as a possible site of NRTI-induced events [34]. All NRTIs caused a rapid and significant ATP depletion of approximately 20% (Fig 3A). The complex IV (cytochrome oxidase) inhibitors, sodium azide and potassium cyanide, caused a similar decline in ATP production (S2 Fig). FLT caused a prompt and significant decrease in oxygen consumption rate (OCR) (Fig 3C). Paraquat and H_2_O_2_ did not induce changes in OCR (S3 Fig). Taken together, these results suggest that exposure to NRTIs caused immediate MRC dysfunction and that this mechanism may precede the swift rise in ROS levels and mtDNA copy number fluctuations.

### Supplementation of antioxidants attenuates the FLT induced ATP decrease

In order to assess whether ROS has an immediate effect on ATP levels we exposed worms to paraquat and H_2_O_2_. Similar to antiretroviral drugs, paraquat and H_2_O_2_ rapidly decreased ATP levels (Fig 3B). It is therefore unclear if NRTIs directly inhibit the MRC or that the production of ROS upon their addition causes MRC inhibition. NAC significantly rescued the ATP level decrease caused by FLT (Fig 3D) and showed a trend towards the rescue of paraquat induced MRC dysfunction (Fig 3E). 500μM NAC did not change ATP levels (S4 Fig). Collectively these results indicate that the prompt decline in MRC function caused by FLT may be instigated by ROS production, although it remains unclear where the origin of these ROS lies.

### NRTIs induce changes in life-history traits

Increased mitochondrial ROS has been found to trigger adaptive responses, culminating in stress resistance and increased longevity in a process known as mitohormesis [35]. We therefore investigated whether the NRTI induced increase in ROS causes a mitohormetic like response and alters life-history traits. The benchmark NRTI, AZT, showed a biphasic dose response confirming mitohormetic effects of NRTIs in *C*. *elegans* (S5 Fig). Exposure of animals from the L1 larval stage to NRTIs significantly extended both mean and maximal lifespan (Table 2). As has been demonstrated before [36], paraquat extended mean lifespan of nematodes exposed from the larval L1 stage (Table 2). Exposure of L4 animals to selected thymidine analogue NRTIs also caused significant mean and maximal lifespan extension. Furthermore, we verified that the NRTI induced lifespan extension is not dependent on caloric restriction (S6 Fig).

**Table 2:** NRTIs caused average lifespan extension. Lifespan extension is dependent on the timing of exposure: A, exposure from larval L1; B, exposure from larval L4. NRTI concentration = 200μM. All animals are N2 at 20°C, mean and maximum refer to the amount of days after L1 (A), and L4 (B). Statistics of NRTIs at L4 were conducted compared their respective DMSO control (AZT, d4T, ddI, & FLT = 0.067%; ddC = 0.2%). All other statistics are compared to controls. SEM = standard error of the mean.

	Exposure	N (total)	Mean ± SEM	Maximum ± SEM	P-value (Mantel-Cox test)	P-value (Gehan-Breslow-Wilcoxon test)
**A (L1)**	Control	183	17.0 (±1.0)	31.0 (±4.2)		
	100μM Paraquat	46	21.0 (±0.0)	31.0 (±0.0)	0.0131	<0.0001
	AZT	249	24.0 (±2.1)	36.7 (±2.9)	<0.0001	<0.0001
	d4T	210	22.0 (±1.0)	36.0 (±2.5)	<0.0001	<0.0001
	ddC	195	20.5 (±2.5)	33.0 (±6.0)	0.0004	0.0330
	ddI	508	20.3 (±2.4)	35.3 (±2.8)	<0.0001	<0.0001
	FLT	391	23.8 (±2.3)	38.8 (±3.7)	<0.0001	<0.0001
**B (L4)**	Control	680	18.0 (±1.1)	27.8 (±1.8)		
	0.067% DMSO	98	18.0 (±4.0)	24.3 (±3.3)	<0.0001	<0.0001
	AZT	164	24.5 (±2.5)	32.5 (±0.5)	<0.0001	<0.0001
	d4T	176	23.3 (±0.7)	31.7 (±1.6)	<0.0001	<0.0001
	FLT	175	19.8 (±2.5)	28.0 (±1.7)	<0.0001	<0.0001

Besides lifespan, fitness is an important determinant of ageing and as a potential proxy of fitness, thrashing rates can be easily measured in *C*. *elegans* [37]. AZT and FLT decreased the thrashing rate at all time points. d4T decreased thrashing rate at 24h but not at later time points. 100μM paraquat also significantly decreased thrashing rates at 24 and 48hrs, yet showed increased thrashing rates at 72hrs (Table 3). With the exception of FLT, all NRTIs slightly yet significantly reduced body length when exposed to L1 or L4 animals. FLT increased body length when exposed to L1 animals and decreased body length when exposed to L4 animals (Table 4).

**Table 3:** NRTIs reduced the number of thrashes per worm per minute. NRTI concentration = 200μM. Statistics were calculated by a two way ANOVA with Dunnett’s multiple comparisons test, compared to the control of that same time point, of 10 worms per treatment condition over ≥3 independent trials. * = P<0.05, ** = P<0.01, *** = P<0.001, n.s. = not significant.

	Control	AZT	d4T	FLT	100μM PQ
**24h**	134.7 (±13.8)	113.9 (±21.7) ***	118.6 (±14.6) ***	119.5 (±13.9) ***	122.5 (±16.2)**
**48h**	125.9 (±15.0)	106.9 (±18.3) ***	123.7 (±17.9) n.s.	103.7 (±15.5) ***	109.5 (±11.6)***
**72h**	111.8 (±13.1)	95.6 (±11.5) ***	109.3 (±12.1) n.s.	89.2 (±108.1) ***	119.6 (±11.8)*

**Table 4:** NRTIs reduced nematode body length. Relative body length was measured after 48hrs NRTI exposure of L1 and 96h exposure of L4 animals (30 worms in ≥2 individual experiments). NRTI concentration = 200μM. Statistics were calculated with a two-way ANOVA with replication, compared to controls. *** = P<0.001.

	Control	AZT	d4T	FLT
**L1**	100 (±13)	87 (±13) ***	92 (±9) ***	106 (±12) ***
**L4**	100 (±11)	95 (±16) ***	93 (±16) ***	95 (±11) ***

### Mitohormetic effects of NRTIs

We reasoned that the NRTI induced increase in ROS would trigger adaptive responses culminating in increased protection to additional stress [20,24]. Exposure of NRTI pre-treated nematodes to 4mM paraquat resulted in increased survival for ddC, ddI and FLT compared to control animals, whereas AZT and d4T showed no change (S7 Fig), indicating a mitohormetic response. Supporting that ROS are driving the mitohormetic response, NAC showed rescue of the FLT induced decrease in fitness after 48h (Table 5). NAC alone did not change thrashing rates (S1 Table). The increase in average lifespan caused by FLT exposure from the larval L4 stage was also mitigated upon co-administration of NAC (Table 6), suggesting that FLT induced ROS production is necessary for lifespan extension.

**Table 5:** Anti-oxidant NAC attenuated the decrease in fitness caused by FLT. Fitness was measured by the number of sigmoidal body bends per worm per minute. FLT concentration = 200μM. Anti-oxidant concentration = 100μM. Statistics were calculated with a two way ANOVA with Turkey’s multiple comparisons test from 10 worms per treatment condition over ≥3 independent trials. FLT compared to control of that same time point. FLT + NAC compared to FLT of that same time point. * = P<0.05, ** = P<0.01, *** = P<0.001, n.s. = not significant.

	Control	FLT	FLT + NAC
**24h**	129.4 (±13.7)	119.5 (±13.9) ***	119.7 (±16.4) n.s.
**48h**	119.5 (±17)	103.7 (±15.5) ***	121 (±9.2) ***
**72h**	107.7 (±16.3)	89.2 (±108.1) ***	108.2 (±17.3) ***

**Table 6:** NAC can mitigate the average lifespan increase caused by FLT. All animals are N2 exposed from L4 at 20°C. Statistics were conducted compared to the DMSO control. FLT concentration = 200μM, NAC concentration = 100μM.

L4	N (total)	Mean ± SEM	Maximum ± SEM	P-value (Mantel-Cox test)	P-value (Gehan-Breslow-Wilcoxon test)
**0.067% DMSO**	107	15.0 (±4.0)	24.3 (±3.3)		
**FLT**	175	19.0 (±2.5)	28.0 (±1.7)	<0.0001	<0.0001
**FLT + NAC**	143	14.0 (±0.3)	28.5 (±4.5)	0.9342	0.6005

### Thymidine analogues induce longevity signalling pathways

NRTI induced MRC inhibition and increased ROS production likely lead to mitohormetic responses that cause lifespan extension similar to the modes of action shown to cause prolonged longevity in *C*. *elegans* mitochondrial (*mit*) mutants [38,39]. It has been suggested that *mit* mutants, which have a dysfunctional MRC, acquire their longevity via an increased production of ROS as a substantial amount of genes are similarly regulated in both *mit* mutants and nematodes exposed to a low dose of paraquat [38]. Besides increased mitochondrial ROS levels and an increase in lifespan, most *mit* mutants show a decrease in fitness and body length [39] similar to nematodes exposed to NRTIs. To attest this hypothesis we performed transcriptome analysis using RNAseq and mapped thymidine analogue differentially expressed genes (DEGs) to pathways which have previously been proposed to be responsible for the lifespan extension seen in *mit* mutants.

The extended lifespan of *clk-1* (ubiquinone production) and *isp-1* (complex III) mutants has been proposed to depend on the hypoxia-inducible transcription factor HIF-1, which is activated by a mild increase in ROS [40]. In this way, HIF-1 links respiratory stress in the mitochondria to a nuclear transcriptional response that promotes longevity [41]. d4T expression profiles had significant overlap with HIF-1 regulated genes at both 24h and 72h (Tables 7 & 8). The *C*. *elegans* p53 homolog, CEP-1, is also known to modulate longevity in *mit* mutants [39]. All thymidine analogues at 24h and 72h induce CEP-1 regulated genes (Tables 7 & 8). This considerable overlap may suggest that CEP-1 longevity signals regulate the life span extension seen in nematodes exposed to thymidine analogues.

**Table 7:** Overlap of thymidine analogue induced DEGs with genes known to be regulated by HIF-1, CEP-1 and SKN-1 after 24h exposure. The expression direction (Up or Down) of thymidine analogue DEGs, with an adjusted P-value <0.01, at 24h, are compared to respectively Up or Down regulated genes from the different conditions taken from [44–4,6]. The numbers of genes that overlap per condition at each time point are given with their representative percentage of thymidine analogue regulated genes between brackets. Over-represented P-value: * = P<0.05, ** = P<0.01, *** = P<0.001, n.s. = not significant. n.a. = not applicable.

		24h					
		AZT		d4T		FLT	
Condition	Expression direction	Down	Up	Down	Up	Down	Up
**HIF-1**	n.a.	2 (0.3%)	1 (0.2%)	3 (0.1%)	20 (0.6%) ***	4 (0.3%)	5 (0.7%)
**CEP-1**	Down	130 (16.7%) **	28 (6.6%)	415 (17.1%) ***	112 (3.5%)	225 (18.8%) ***	28 (3.8%)
	Up	5 (0.6%)	69 (16.3%) ***	59 (2.4%)	616 (19.4%) ***	26 (2.2%)	229 (30.7%) ***
**SKN-1 (ALL)**	Down	1 (0.1%)	2 (0.5%)	9 (0.4%)	16 (0.5%)	4 (0.3%)	5 (0.7%)
	Up	5 (0.6%)	18 (4.2%) ***	21 (0.9%)	71 (2.2%) ***	5 (0.4%)	31 (4.2%) ***

**Table 8:** Overlap of thymidine analogue induced DEGs with genes known to be regulated by HIF-1, CEP-1 and SKN-1 after 72h exposure. The expression direction (Up or Down) of thymidine analogue DEGs, with an adjusted P-value <0.01, at 72h, are compared to respectively Up or Down regulated genes from the different conditions taken from [44–46]. The numbers of genes that overlap per condition at each time point are given with their representative percentage of thymidine analogue regulated genes between brackets. Over-represented P-value: * = P<0.05, ** = P<0.01, *** = P<0.001, n.s. = not significant. n.a. = not applicable.

		72h					
		AZT		d4T		FLT	
Condition	Expression direction	Down	Up	Down	Up	Down	Up
**HIF-1**	n.a.	5 (0.2%)	12 (0.3%)	4 (1.2%) *	2 (0.5%)	2 (0.4%)	5 (0.9%) *
**CEP-1**	Down	618 (20.4%) ***	116 (3.4%)	41 (12.1%)	10 (2.3%)	97 (20.3%) ***	16 (2.7%)
	Up	50 (1.7%)	604 (17.6%) ***	25 (7.4%)	72 (16.6) ***	20 (4.2%)	74 (12.7%) *
**SKN-1 (ALL)**	Down	11 (0.4%)	9 (0.3%)	3 (0.9%)	1 (0.2%)	5 (1%) *	3 (0.5%)
	Up	13 (0.4%)	86 (2.5%) ***	7 (2.1%)	8 (1.8%)	11 (2.3%)	7 (1.2%)

Oxidative stress responses are frequently governed by the redox sensitive transcription factor SKN-1. SKN-1 also acts in multiple longevity pathways [39,42,43]. At 24h, all NRTIs showed expression profiles that overlap with SKN-1 up-regulated genes, suggesting a role for ROS in the longevity response (Table 7). At 72h the overlap remained for AZT, but was absent for d4T. FLT at 72h showed a small yet significant overlap with SKN-1 down-regulated genes (Table 8). Taken together, our observations suggest that NRTI induced MRC inhibition and increased ROS production in *C*. *elegans* lead to mitohormetic responses that signal lifespan extension.

## Discussion

**Fig 4:**
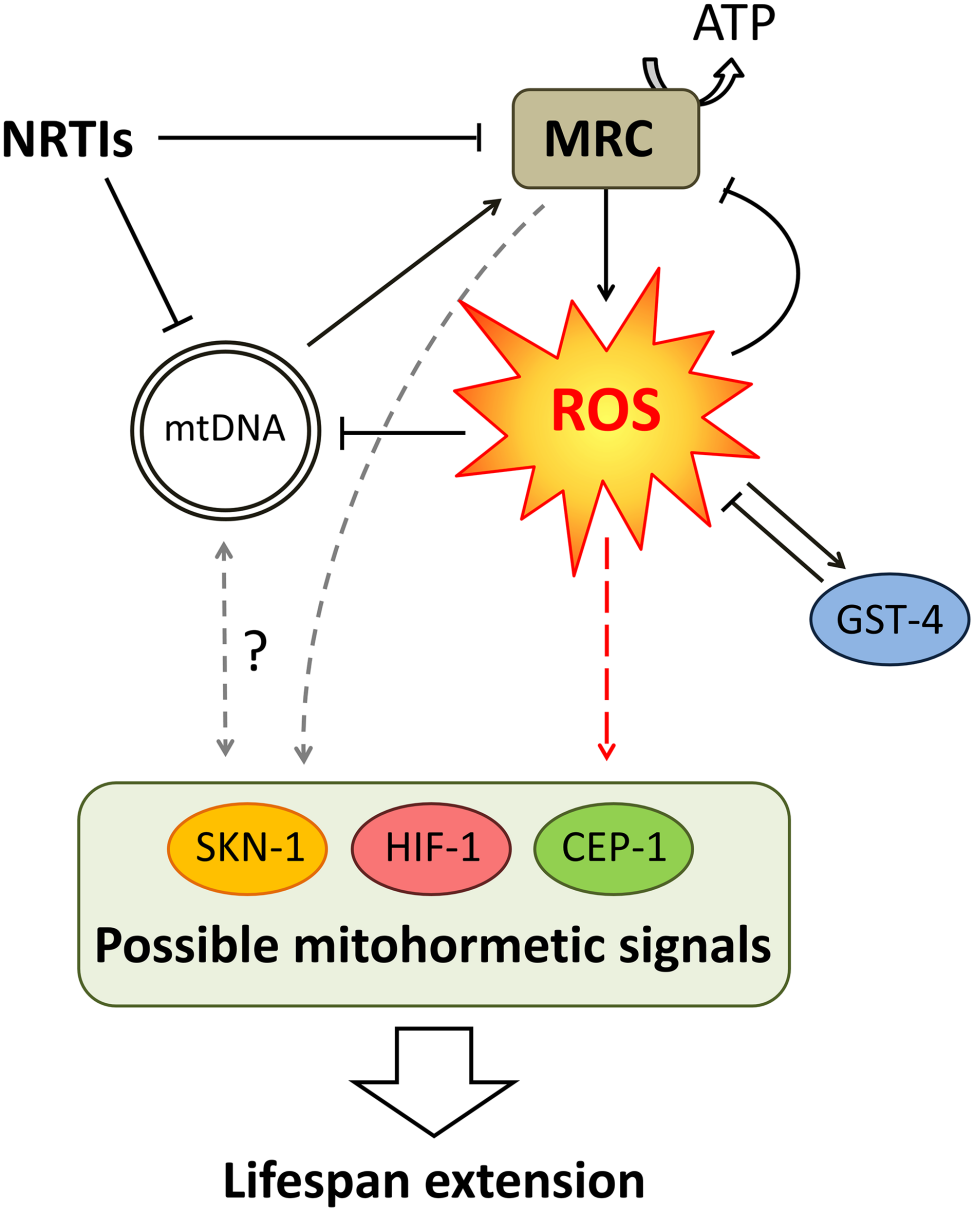
Schematic representation of short-term NRTI induced events which lead to longevity in *C*. *elegans*. NRTIs directly inhibit the mitochondrial respiratory chain (MRC) inducing an increase in reactive oxygen species (ROS). NRTIs also inhibit mtDNA replication which can, in time, disrupt MRC function through a depletion of mtDNA encoded transcripts and MRC complex components. Augmented ROS production likely induces mitohormetic signalling which involves factors involved in prolonged longevity signalling culminating in increased lifespan (red dashed arrow). Based on our results, likely signalling pathway include CEP-1, SKN-1 and HIF-1. NRTI induced ROS also prompts *gst-4* expression. Black solid arrows indicate results from this study. Grey dashed arrows indicate hypothetical pathways.

In this study we show that exposure of *C*. *elegans* to NRTIs can rapidly change mtDNA copy number, decrease ATP levels and increase the production of ROS, all within a time frame too short for the causality of events as proposed in the polymerase-γ theory to take place. The ability of paraquat or H_2_O_2_ to induce similar effects in both mtDNA copy numbers and MRC inhibition, suggests that increased ROS production is leading in these changes. Supporting this, the antioxidant NAC attenuated the short-term decrease in mtDNA copy number and ATP levels caused by FLT. Furthermore we show that the FLT induced reduction in fitness and increase in lifespan can be attenuated by NAC, suggesting that oxidative stress is an important driver behind NRTI induced adverse events. Signalling pathways governed by CEP-1 and HIF-1 are likely induced upon MRC perturbation and SKN-1 likely responds to the upsurge in ROS, collectively leading to mitohormetic events (Fig 4).

### NRTIs induce immediate mitochondrial toxicity

NRTI induced mitochondrial toxicity is commonly discussed in the context of chronic exposure. We discovered that NRTIs cause rapid and unexpected changes in mtDNA copy numbers. The prompt rise in mtDNA copy number induced by AZT was particularly surprising and may be caused by complex I inhibition [31–33]. This is supported by the discovery that inhibition of complex I by rotenone significantly increased mtDNA levels [47]. The d4T induced rise in mtDNA copy number may also be caused by inhibition of complex I, which has been observed after chronic exposure [31]. MtDNA copy number fluctuations may occur due to the NRTI induced increase in ROS production as mild oxidative stress is known to prompt mitochondrial biogenesis [48].

The sharp decrease in mtDNA copy number upon exposure to paraquat and the partial rescue of FLT decreased mtDNA copy number upon supplementation with NAC suggest, however, that ROS production can also restrain mtDNA replication. Polymerase-γ activity can be reduced due to oxidative damage [16]. Taken together, we conclude that the rapid fluctuations in mtDNA copy number upon exposure to NRTIs are strongly affected by the generation of ROS. However, the findings that NAC supplementation does not completely rescue the FLT-induced mtDNA copy number decline suggests that there are other mechanisms at play. For instance, NRTI metabolism and pharmacokinetics likely interfere with normal nucleoside homeostasis and function [12], and NRTIs can also directly inhibit mitochondrial enzymes besides polymerase-γ [13] (for a comprehensive review see [4]).

### Rapid NRTI induced inhibition of MRC function

ROS can be generated via MRC inhibition [49]. NRTIs, in particular AZT, have been suggested to directly inhibit the mitochondrial respiratory chain [33,50]. Inhibition of complex I by AZT has been proposed to increase NADH derived electrons which are consequently diverted from the MRC to alternate electron acceptors such as oxygen, thus stimulating free radical generation [31,32]. ddC has been shown to directly inhibit complex I through phosphorylation of complex I at Q-module subunits [32]. It is therefore expected that AZT and ddC cause direct MRC dysfunction and consequently increase ROS production.

ROS are also known to rapidly inhibit MRC complexes and decrease ATP levels [51,52]. We suggest, however, that FLTs reduction in MRC function is probably first caused by direct MRC inhibition as NAC supplementation did not unequivocally rescue the diminished ATP levels. This may indeed be the case for all NRTIs, as it is unclear how they can so quickly cause ROS production without first inhibiting the MRC. Moreover, our observations that NAC readily rescues the decreased ATP levels caused by paraquat, which is not known to directly inhibit MRC complex function at the concentrations used in this study supports this proposed chronology of events [53,54].

Paraquat is known to generate ROS in the mitochondrial matrix at the site of complex I [53]. The inhibition of the MRC by NRTIs probably also results in generation of ROS within the mitochondrial matrix [55] as we previously showed that all NRTIs reduced quinone redox status [25]. On the basis of structural features alone, NRTIs likely effectively compete with the nicotinamide adenine dinucleotide (NAD) recognition site of complex I [31]. Complex I has many suspected nucleotide binding sites [32], making it the most likely of MRC complexes to interact with NRTIs. The similarity of NRTI induced adverse events to symptoms observed in inherited mitochondrial disorders that specifically affect NADH binding sites, such as the early onset of neurodegenerative disorders, lactic acidosis, cardiomyopathy, and exercise intolerance, also supports this. Moreover, mutations in the complex I NADH binding sites are known to increase ROS production [56].

ROS likely also induce a decline in ATP levels through inhibition of the adenine nucleotide translocase which has been observed to be the protein in mitochondrial membranes most sensitive to oxidative damage and consequent decline in activity [52]. Superoxide has also been found to rapidly decrease ATP levels by reducing mitochondrial membrane potential (ΔΨ_mt_) through activation of uncoupling proteins. It has been suggested that in this way, increased superoxide production by the MRC activates a feedback-loop designed to mitigate ROS production [57]. We therefore propose that NRTIs directly inhibit the MRC and cause an initial rise in ROS which rapidly inhibits MRC function further. More research is, however, necessary to unravel the sites where NRTIs might inhibit MRC complexes.

### NRTIs induce a mitohormetic lifespan extension in *C*. *elegans*

A retarded development and reduced body length in *C*. *elegans* is often related to an increase in lifespan. *C*. *elegans* long-lived *mit* mutants, for instance, display a spectrum of phenotypes that are typically unified by reduced rates of development, ageing and behaviour [39], similar to the phenotypes witnessed here with NRTIs. In line with this, disruption of almost any subunit of the MRC, regulators of its assembly, and its co-factors, can lead to lifespan extension in *C*. *elegans* [58]. Low doses of mitochondrial toxins, such as rotenone for instance, are known to increase longevity [39]. Interestingly, impaired respiration as seen in *mit* mutants was associated with an increase in mtDNA copy numbers, similar to our observations for NRTIs (Fig 1A). We propose that in *C*. *elegans*, NRTI induced MRC inhibition and ROS production prompt mitohormetic signalling pathways similar to those seen in long-lived *mit* mutants. It has been suggested that *C*. *elegans mit* mutants acquire their longevity via an increased production of ROS as a substantial amount of genes are similarly regulated in both *mit* mutants and nematodes exposed to a low dose of paraquat [38]. GST-4 has been shown to be directly involved in resistance to oxidative stress [26] and increased *gst-4* expression has been found in *mit* mutants as part of the ROS induced ‘retrograde response’ [59]. In agreement, our observed up-regulation of *gst-4* suggests that a defence response is induced by ROS upon exposure to NRTIs. Additionally, we show that pre-exposure to NRTIs protects animals from paraquat stress and extends lifespan; both consequences of mitohormesis. Finally, the extended lifespan of *clk-1* and *isp-1* mutants, and RNAi knock-down of many MRC components, depends on CEP-1, HIF-1 and SKN-1 [40,58], which showed significant overlap with DEGs induced by NRTI exposure.

One protective strategy to limit mitochondrial oxidative damage and consequent cellular injury is the use of antioxidants [60,61]. Indeed, this strategy has been tested both *in vitro* [62,63] and *in vivo* [62,64,65] as a means to protect mitochondrial function, alleviate HAART or HIV-1 induced adverse events, and boost the patient’s immune system [65–70]. NAC has even been supported in becoming a standard complementary treatment to antiretroviral therapy [71]. The inability of NAC to completely rescue FLT reduced fitness and mtDNA depletion shows that besides MRC dysfunction and ROS production, NRTI induced mtDNA replication stalling by polymerase-γ inhibition may also influence the observed changes in life-history traits. This is especially the case for FLT that showed severely decreased mtDNA copy number. This effect, however, is probably small for the other NRTIs as a 55% reduction in mtDNA copy number in *C*. *elegans* has recently been shown to have limited influence on life history traits [72].

### Drug concentrations and exposure

We selected a NRTI concentration range of 100–200μM on the basis of their ability to cause robust effects on mitochondrial function in *C*. *elegans* [25]. In HIV-1 patients, the plasma concentration of the antiretroviral drugs used in this study is generally between 4–16μmol/L [73]. However, plasma concentrations of 50μM have been observed for AZT in the clinic [32]. *In vitro* experiments commonly use concentrations between 10–200μM [11,74,75]. Drugs can be exposed to nematodes on solid media, by either mixing them in with the bacterial lawn or adding them directly to the agar plates [76,77]. We found both methods to be effective, yet preferred mixing the drugs into the agar plates so as to limit drug diffusion over time. In this way, there are two ways in which the antiretrovirals can reach their targets, namely through ingestion or by diffusion across the nematodes cuticle. The cuticle of *C*. *elegans* is notoriously impervious to many compounds and drug uptake by *C*. *elegans* is rather poor. Moreover, *C*. *elegans* has extensive enzymatic xenobiotic defences and exogenously applied pharmacologicals that do penetrate the cuticle often fail to accumulate to effective concentrations within tissues which may explain the rapid adaptation of the GST-4 expression in *C*. *elegans* [78]. It is therefore not uncommon for polar drugs to be applied to *C*. *elegans* in a concentration 1000 fold higher than their predicted affinity for the target [79]. More research is however needed as although the drug concentrations used in this study have been chosen with care, their ability to cause similar effects in patients needs to be verified. Of particular interest is that NRTI-induced adverse events are known to be dependent on the NRTIs phosphorylation state [80,81]. The detection of NRTI-TPs, however, in patients is difficult and has limited pharmacological research into the pharmacokinetics of NRTIs [82]. Measurements of NRTI nucleoside and nucleotide concentrations within nematodes may help explain discrepancies between the toxicity profiles of each NRTI and shed light on the pharmacokinetics of each compound.

## Conclusions

Taken together, this study shows that ROS production in the initial stages of NRTI treatment may strongly influence the progression of disease and that palliative measures to reduce ROS early on may prove effective in reducing the occurrence NRTI adverse events. During chronic exposure to NRTIs, the increased production of ROS together with the sustained inhibition of polymerase-γ may tip the balance in the favour of mitochondrial toxicity, predisposing the patient to the onset of adverse events. It is therefore perhaps not surprising that there is a high occurrence of ROS related inflammation and metabolic syndromes amongst the HIV-1 infected being treated with NRTIs [17].

## Materials & methods

### Nematode culture and drug exposure

Nematodes were cultured on NGM plates and fed OP50 *Escherichia coli* lawn at 20°C unless mentioned otherwise. The N2 Bristol (wild type) and MJCU017 strains were provided by the Caenorhabditis Genetics Center (CGC) and PE255 animals were a generous gift from C. Lagido. *Gst-4* expression measurements were performed using ‘JMET69’ (Is[gst-4(1491 bp)::gfp;unc119(+)];Is[unc-54pro::mCherry;unc119(+)]), which was generated using MJCU017 [83].

Antiretroviral drugs (Sigma-Aldrich Co.) were dissolved in dimethyl sulfoxide (DMSO) (AZT, FLT, d4T & ddI = 300mM; ddC = 100mM). Antioxidant (Sigma-Aldrich Co.) *N*-acetylcysteine (NAC) was dissolved to a stock solution of 500mM in ddH_2_O.

### RNA extraction, library construction and sequencing

In biological triplicates, synchronized L1 larvae were placed on NGM plates with an OP50 bacterial lawn until L4, and transferred to plates containing the drug of interest for 24 or 72 hours. A final concentration of 100μM was added to the NGM media before pouring. Besides unexposed nematodes, a representative DMSO concentration (0,033%) was used as a control. Bacteria and eggs were removed from the culture using a 31μM pore mesh before immediately freezing the samples at -80°C until RNA extraction. 72 hour cultures were moved to fresh plates every day after meshing. RNA was extracted from worm pellets using the Direct-zol RNA MiniPrep kit (Zymo Research, R2052) as described by the manufacturer. RNA integrity and quality were analyzed using the Agilent 2100 Bioanalyser (G2938A) before Illumina Sequencing (BaseClear B.V., Leiden, The Netherlands). Sequencing was carried out using the Illumina TruSeq library preparation kit, sequencing with 20 million reads per sample.

### RNAseq data processing and statistics

FastQC (http://www.bioinformatics.babraham.ac.uk/projects/fastqc) was used to check the quality of the raw reads. No samples were omitted from statistical analysis because of bad quality. The sequence reads were trimmed using Trimmomatic v0.32 [84]. The trimmed reads were aligned against the *C*. *elegans* genome (Ensembl, WBcel235.75) using TopHat (v2.0.8) [85]/Bowtie (v2.1.0) [86]. HTseq [87] was used to quantify gene expression. Statistical analysis was performed in R/Bioconductor, and the DESeq package [88] was applied for differential gene expression analysis.

### Paraquat stress assays

Paraquat sensitivity was tested as described previously [89,90]. In short, L4 larvae were exposed to NRTIs until their larvae had reached the L4 stage. These were then transferred to NGM plates containing 4mM paraquat and kept for 72hrs at 20°C, at which time the number of surviving worms was calculated. Paraquat sensitivity from L4 animals was tested by exposing synchronized animals to NRTIs from the L1 larval stage until L4. These were then transferred to NGM plates containing 4mM paraquat, kept at 20°C and the number of surviving worms was counted approximately every other day. Results were combined from at least two independent experiments.

### ROS measurements

GFP expression, driven by the *gst-4* promoter, was measured after 24h culture of L4 JMET69 animals in the presence of NRTIs. Short-term experiments (6h) were performed on therapy naïve animals. Synchronized worms were cultured at 25°C to L4, transferred to FUdR plates until day 1 of adulthood and washed from the plates with M9 buffer and filtered over a SEFAR NITEX^®^ 31μM pore mesh (03-31/24) to remove debris and *E*. *coli*. ~800 animals were placed in each well of a flat clear-bottomed 96 well plate and exposed to 200μM of NRTIs in M9 buffer, in a total volume of 100μL. Measurements were performed at 25°C and recorded by a FLUOstar OPTIMA plate reader (Biotek Synergy Mx). GFP (470/520nm) expression was normalized to constitutively active RFP (577/620nm) expression. Statistical analysis compared to DMSO: AZT, FLT, d4T & ddI = 0.067%; ddC = 0.2%. Experiments were performed with a minimum of three biological and five technical replicates.

### ATP measurements

ATP luminescence measurements were adapted from [91]. All measurements were performed in CPB (pH 6.5) with a final volume of 100μL. 10μL of a 10x concentrated drug solution (in DMSO) was pipetted into white flat-bottomed 96-wells plates (Greiner). Each condition was measured in at least 3 biological replicates and at least 8 technical replicates. NAC was dissolved in MiliQ. Luminescence was measured in a Biotek Synergy MX plate reader in the visible spectral range (300-600nm). Young adult worms grown at 25°C were washed off the FUdR plates with S-basal and collected in CPB. Luminescence buffer was prepared with 1mM D-Luciferin and 0,05% Triton-X, to improve cuticle permeability. For the NRTIs, the final concentration of DMSO was 0.33% (all final concentrations, in CPB). Luminescence was measured continuously during 2 minutes. The average of green fluorescent protein (GFP) expression was measured continuously with a 485/520nm filter set for 1 minute (12 times) right after the luminescence measurement for normalisation. The different biological replicates were analysed with a two-way ANOVA with replication with a significance value of p ≤ 0,05. The mean ± standard error is reported.

### Oxygen consumption rate

Oxygen consumption was measured using the Seahorse XF^e^96 (Seahorse Bioscience). 20 L4 animals were picked from NGM plates and transferred in 96-well Seahorse plates containing dH_2_O. 10x concentrated compounds were injected into the wells after 4 basal measurements, which were used for normalisation. Oxygen consumption was measured in at least 5 replicates. Data was analysed using Wave 2.2.0 (Seahorse Bioscience).

### Lifespan assays

Lifespan scoring was conducted at 20°C. ~100 synchronized nematodes were placed on NGM plates containing the compound(s) of interest and dead animals were scored approximately every other day. Worms that crawled off the plates, showed bagging or gonad protrusion were removed from the plates and scored separately. Animals were scored as dead when they no longer moved or showed pharyngeal pumping when they were successively gently prodded on the head and tail with a platinum wire. All experiments using L4 animals were done in the presence of 50μM fluorodeoxyuridine (FUdR) to inhibit progeny. A minimum of 2 biological replicates per condition was analysed. Prism 6 software was used for statistical analysis using the log-rank (Mantel-Cox and Gehan-Breslow-Wilcoxon) method [92].

### Thrashing assay

A single worm was placed in a drop of M9 buffer on a clean glass slide and allowed to acclimatize for 30 seconds. The frequency of sigmoidal body bends was counted during 30 seconds as described previously [93]. Thrashes were averaged from 10 worms per treatment condition over three independent trials.

### Body length

Animals were exposed to NRTIs from L1-L4 (48h) or from L4-D3 (96h), washed with M9, and imaged with a Canon Power Shot A640 camera attached to a Leica M80 stereo microscope. Measurements were performed with Image J freeware (W.S. Rasband, U.S.A. National Institutes of Health, Bethesda, Maryland, USA, http://rsb.info.nih.gov/ij/, 1997–2012). Measurements were performed upon at least 30 worms from three independent experiments.

### mtDNA copy number quantitative real time PCR

Quantitative real time PCR was performed as described by de Boer [25]. In short, synchronized N2, 2h post L4 molt, young adult worms were transferred to OP50 seeded FUdR NGM plates containing NRTIs (200μM) or paraquat (100μM, 500μM, 1mM). Five adult worms were collected at predetermined time points during drug exposure and lysed in Lysis buffer (50mM KCl, 10mM Tris (ph 8.3), 2,5mM MgCl_2_, 0,45% NP-40 (IGEPAL), 0,45% Tween-20, 0,01% Gelatin, 20mg/mL Proteinase K). Before detection in the PCR, the solution was diluted 40 times and 2μl was used as input in the PCR reaction. Primers specific for cytochrome c oxidase subunit I (COX1) were used for the determination of mtDNA copy number. PCRs were performed using the Taqman^®^ universal cycling conditions with amplified products being detected using a Taqman^®^ probe for CeCOX1. Fluorescent signal intensities were determined using the 7300 Real-Time PCR System (Applied Biosystems) with software SDS (version 1.9.1). To quantify the absolute quantity of mtDNA per worm, a standard curve was generated from a plasmid with a fragment of the *cox1* gene. After PCR the total mtDNA copies per worm were calculated. mtDNA quantitative PCR was performed with at least three biological and two technical replicates.

## Supporting information

S1 Fig NAC decreased mtDNA replication associated with development. NAC reduced the normal mtDNA copy number increase during 1h exposure. 100μM NAC decrease mtDNA copy number compared to control nematodes after 1h exposure (dark grey vs 1h control). mtDNA copy number increases during nematode development and therefore rises (control 0h vs control 1h). Error bars show the 95% C.I. (df = 51). Significance was determined using a two-tailed student’s T-test assuming unequal variances. ** = P-value <0.01, *** = P-value <0.001 compared to control animals at 1h, and Control 1h vs Control 0h.
(TIF)

S2 Fig Sodium azide and potassium cyanide reduced ATP production. Complex IV inhibitors, sodium azide (NaN3) and potassium cyanide (KCn), reduced ATP production. 1mM NaN3 and 50mM KCn reduced *in vivo* ATP levels measured after 2 minutes of exposure. Statistics were calculated with a two-way ANOVA with replication, compared to the control. * = P<0.05, *** = P<0.001.
(TIF)

S3 Fig H_2_O_2_ and paraquat do not influence OCR. Immediate OCR rates after injection of H_2_O_2_ and paraquat (PQ) do not change. The lack of these pro-oxidants to have an immediate effect on OCR supports the observations that they do not directly inhibit MRC complex function at the concentrations used in this study [53,54].
(TIF)

S4 Fig NAC does not alter ATP levels. ATP levels did not change upon exposure to 500μM NAC. *In vivo* ATP levels measured after 2 minutes of exposure.
(TIF)

S5 Fig AZT portrays a biphasic dose response in *C*. *elegans*. In accordance with mitohormetic events triggered by inhibited MRC function and an increase in ROS, 200μM AZT caused significant lifespan extension in *C*. *elegans*. An increase in AZT concentration above 200μM steadily decreased life span in L4 animals, indicating that NRTIs cause a biphasic dose response, reconfirming hormesis [94]. Increasing the concentration of AZT above a threshold of approximately 200μM reversed the life extension seen with 200μM. Animals were exposed from the L4 larval stage at 20°C. Asterisks indicate significance as calculated with the Mantel-Cox test compared to the control: * = P<0.05, ** = P<0.01, *** = P<0.001, n.s. = not significant.
(TIF)

S6 Fig NRTIs likely did not cause lifespan extension through caloric restriction. In *C*. *elegans*, caloric restriction during development or adulthood can cause lifespan extension by increasing mitochondrial activity and prompting the expression of genes involved in stress resistance and longevity [95]. Although pharyngeal pumping is not a direct measure of feeding we tested if the changes in lifespan traits we observed were caused by caloric restriction due to a reduced food intake (reduced pharyngeal pumping). We exposed nematodes from the L4 larval stage and counted pharyngeal pumping rates at 2, 6, and 24h. No significant change in pumping rate was seen at time points 2, 6, and 24h. Taken together, these results suggest that the observed extension in lifespan is likely not caused by caloric restriction. A: 2h exposure, B: 6h exposure, and C: 24h exposure to NRTIs does not alter pharyngeal pumping rates per minute (ppm) per worm. NRTI concentration = 200μM. L4 animals were exposed to NRTIs and pharyngeal pumping rates of 10 animals from each condition were manually counted under a Leica M80 stereo microscope for the duration of 30 seconds. To assess short-term and long-term effects, drug exposure times were 2, 6, 24, and 48h. Dead and non-pumping animals were not included. 1 pump was defined as 1 grinder movement. Pumping rates were measured at room temperature (approximately 22°C) on NGM agar with confluent lawns of OP50 bacteria.
(TIF)

S7 Fig NRTIs pre-exposure protected against subsequent paraquat exposure. 4mM paraquat (PQ) survival assay of NRTI exposed animals. Statistics were calculated by two sided student’s *t*-test assuming unequal variance, compared to the control of that same time point (72h of PQ exposure).
(TIF)

S1 Table Supplementation of NAC does not affect movement. The number of sigmoidal body bends per worm per minute can be used as a proxy for fitness. Anti-oxidant concentration = 100μM. Statistics were calculated by two sided student’s t-test assuming unequal variance compared to control of that same time point. n.s. = not significant.
(DOCX)

## References

[1] Smith RL, de Boer R, Brul S, Budovskaya Y, van Spek H. Premature and accelerated aging: HIV or HAART? Front Genet. 2012 1;3(January):328. 23372574 10.3389/fgene.2012.00328 PMC3556597

[2] Lewis W, Day BJ, Copeland WC. Mitochondrial toxicity of NRTI antiviral drugs: an integrated cellular perspective. Nat Rev Drug Discov. 2003 10;2(10):812–22. doi: 10.1038/nrd1201 14526384 10.1038/nrd1201

[3] Apostolova N, Blas-García A, Esplugues J V. Mitochondrial toxicity in HAART: an overview of in vitro evidence. Curr Pharm Des. 2011 1;17(20):2130–44. 21718249 10.2174/138161211796904731

[4] Apostolova N, Blas-García A, Esplugues J V. Mitochondrial interference by anti-HIV drugs: mechanisms beyond Pol-γ inhibition. Trends Pharmacol Sci. 2011 12;32(12):715–25. doi: 10.1016/j.tips.2011.07.007 21899897 10.1016/j.tips.2011.07.007

[5] Côté HCF. Possible ways nucleoside analogues can affect mitochondrial DNA content and gene expression during HIV therapy. Antivir Ther. 2005;10 Suppl 2(SUPPL. 2):M3–11. 16152702

[6] Cihlar T, Ray AS. Nucleoside and nucleotide HIV reverse transcriptase inhibitors: 25 years after zidovudine. Antiviral Res. 2010 1;85(1):39–58. doi: 10.1016/j.antiviral.2009.09.014 19887088 10.1016/j.antiviral.2009.09.014

[7] Stankov M V, Lücke T, Das AM, Schmidt RE, Behrens GMN. Mitochondrial DNA depletion and respiratory chain activity in primary human subcutaneous adipocytes treated with nucleoside analogue reverse transcriptase inhibitors. Antimicrob Agents Chemother. 2010 1;54(1):280–7. doi: 10.1128/AAC.00914-09 19805555 10.1128/AAC.00914-09 PMC2798495

[8] Trifunovic A, Wredenberg A, Falkenberg M, Spelbrink JN, Rovio AT, Bruder CE, et al Premature ageing in mice expressing defective mitochondrial DNA polymerase. Nature. 2004 5 27;429(6990):417–23. doi: 10.1038/nature02517 15164064 10.1038/nature02517

[9] Mallon PWG, Unemori P, Sedwell R, Morey A, Rafferty M, Williams K, et al In vivo, nucleoside reverse-transcriptase inhibitors alter expression of both mitochondrial and lipid metabolism genes in the absence of depletion of mitochondrial DNA. J Infect Dis. 2005 5 15;191(10):1686–96. doi: 10.1086/429697 15838796 10.1086/429697

[10] Viengchareun S, Caron M, Auclair M, Kim MJ, Frachon P, Capeau J, et al Mitochondrial toxicity of indinavir, stavudine and zidovudine involves multiple cellular targets in white and brown adipocytes. Antivir Ther. 2007 1;12(6):919–29. 17926646

[11] Pan-Zhou XR, Cui L, Zhou XJ, Sommadossi JP, Darley-Usmar VM. Differential effects of antiretroviral nucleoside analogs on mitochondrial function in HepG2 cells. Antimicrob Agents Chemother. 2000 3;44(3):496–503. 10681309 10.1128/aac.44.3.496-503.2000 PMC89717

[12] Hosseini SH, Kohler JJ, Haase CP, Tioleco N, Stuart T, Keebaugh E, et al Targeted transgenic overexpression of mitochondrial thymidine kinase (TK2) alters mitochondrial DNA (mtDNA) and mitochondrial polypeptide abundance: transgenic TK2, mtDNA, and antiretrovirals. Am J Pathol. 2007 3;170(3):865–74. doi: 10.2353/ajpath.2007.060655 17322372 10.2353/ajpath.2007.060655 PMC1864875

[13] Valenti D, Barile M, Passarella S. AZT inhibition of the ADP/ATP antiport in isolated rat heart mitochondria. Int J Mol Med. 2000 7;6(1):93–6. 10851273 10.3892/ijmm.6.1.93

[14] Blas-Garcia A, Apostolova N, Esplugues J V. Oxidative stress and mitochondrial impairment after treatment with anti-HIV drugs: clinical implications. Curr Pharm Des. 2011 12 1;17(36):4076–86. 22188456 10.2174/138161211798764951

[15] Day BJ, Lewis W. Oxidative stress in NRTI-induced toxicity: evidence from clinical experience and experiments in vitro and in vivo. Cardiovasc Toxicol. 2004 1;4(3):207–16. 15470269 10.1385/ct:4:3:207

[16] Graziewicz M a, Day BJ, Copeland WC. The mitochondrial DNA polymerase as a target of oxidative damage. Nucleic Acids Res. 2002 7 1;30(13):2817–24. 12087165 10.1093/nar/gkf392 PMC117047

[17] Trifunovic A, Larsson N-G. Mitochondrial dysfunction as a cause of ageing. J Intern Med. 2008 2;263(2):167–78. doi: 10.1111/j.1365-2796.2007.01905.x 18226094 10.1111/j.1365-2796.2007.01905.x

[18] Kline ER, Bassit L, Hernandez-Santiago BI, Detorio MA, Liang B, Kleinhenz DJ, et al Long-term exposure to AZT, but not d4T, increases endothelial cell oxidative stress and mitochondrial dysfunction. Cardiovasc Toxicol. 2009 3;9(1):1–12. doi: 10.1007/s12012-008-9029-8 19067249 10.1007/s12012-008-9029-8 PMC2714048

[19] Manda KR, Banerjee A, Banks W a, Ercal N. Highly active antiretroviral therapy drug combination induces oxidative stress and mitochondrial dysfunction in immortalized human blood-brain barrier endothelial cells. Free Radic Biol Med. 2011 4 1;50(7):801–10. doi: 10.1016/j.freeradbiomed.2010.12.029 21193030 10.1016/j.freeradbiomed.2010.12.029 PMC5997409

[20] Ristow M. Unraveling the Truth About Antioxidants: Mitohormesis explains ROS-induced health benefits. Nat Med. 2014 7;20(7):709–11. doi: 10.1038/nm.3624 24999941 10.1038/nm.3624

[21] Sena L a, Chandel NS. Physiological roles of mitochondrial reactive oxygen species. Vol. 48, Molecular Cell. Elsevier Inc.; 2012 p. 158–66. 10.1016/j.molcel.2012.09.025 PMC3484374 23102266

[22] D’Autréaux B, Toledano MB. ROS as signalling molecules: mechanisms that generate specificity in ROS homeostasis. Nat Rev Mol Cell Biol. 2007 10;8(10):813–24. doi: 10.1038/nrm2256 17848967 10.1038/nrm2256

[23] Mandas A, Iorio EL, Congiu MG, Balestrieri C, Mereu A, Cau D, et al Oxidative imbalance in HIV-1 infected patients treated with antiretroviral therapy. J Biomed Biotechnol. 2009 1;2009:749575 doi: 10.1155/2009/749575 19884983 10.1155/2009/749575 PMC2768042

[24] Segref A, Kevei É, Pokrzywa W, Schmeisser K, Mansfeld J, Livnat-Levanon N, et al Pathogenesis of human mitochondrial diseases is modulated by reduced activity of the ubiquitin/proteasome system. Cell Metab. 2014 4 1;19(4):642–52. doi: 10.1016/j.cmet.2014.01.016 24703696 10.1016/j.cmet.2014.01.016

[25] de Boer R, Smith RL, de Vos WH, Manders EM, Brul S, van der Spek H. Caenorhabditis elegans as a model system to study drug induced mitochondrial toxicity. PLoS One. 2015;10(5):1–16. 10.1371/journal.pone.0126220 PMC4430419 25970180

[26] Leiers B, Kampkötter A, Grevelding CG, Link CD, Johnson TE, Henkle-Dührsen K. A stress-responsive glutathione S-transferase confers resistance to oxidative stress in Caenorhabditis elegans. Free Radic Biol Med. 2003 6;34(11):1405–15. 12757851 10.1016/s0891-5849(03)00102-3

[27] Xue SY, Hebert VY, Hayes DM, Robinson CN, Glover M, Dugas TR. Nucleoside Reverse Transcriptase Inhibitors Induce a Mitophagy-Associated Endothelial Cytotoxicity That Is Reversed by Coenzyme Q10 Cotreatment. Toxicol Sci. 2013 5 2;134(2):323–34. doi: 10.1093/toxsci/kft105 23640862 10.1093/toxsci/kft105 PMC3842105

[28] Tsang WY, Lemire BD. Mitochondrial genome content is regulated during nematode development. Biochem Biophys Res Commun. 2002 2 15;291(1):8–16. doi: 10.1006/bbrc.2002.6394 11829454 10.1006/bbrc.2002.6394

[29] Bratic I, Hench J, Henriksson J, Antebi A, Bürglin TR, Trifunovic A. Mitochondrial DNA level, but not active replicase, is essential for Caenorhabditis elegans development. Nucleic Acids Res. 2009 4;37(6):1817–28. doi: 10.1093/nar/gkp018 19181702 10.1093/nar/gkp018 PMC2665216

[30] Martin JL, Brown CE, Matthews-Davis N, Reardon JE. Effects of antiviral nucleoside analogs on human DNA polymerases and mitochondrial DNA synthesis. Antimicrob Agents Chemother. 1994 12;38(12):2743–9. 7695256 10.1128/aac.38.12.2743 PMC188279

[31] Lund KC, Wallace KB. Direct, DNA pol-gamma-independent effects of nucleoside reverse transcriptase inhibitors on mitochondrial bioenergetics. Cardiovasc Toxicol. 2004 1;4(3):217–28. 15470270 10.1385/ct:4:3:217

[32] Lund KC, Wallace KB. Adenosine 3’,5’-cyclic monophosphate (cAMP)-dependent phosphoregulation of mitochondrial complex I is inhibited by nucleoside reverse transcriptase inhibitors. Toxicol Appl Pharmacol. 2008 1 1;226(1):94–106. doi: 10.1016/j.taap.2007.08.015 17904600 10.1016/j.taap.2007.08.015 PMC2390784

[33] Pereira LF, Oliveira MBM, Carnieri EGS. Mitochondrial sensitivity to AZT. Cell Biochem Funct. 1998;16(3):173–81. doi: 10.1002/(SICI)1099-0844(199809)16:3<173::AID-CBF783>3.0.CO;2-4 9747509 10.1002/(SICI)1099-0844(199809)16:3<173::AID-CBF783>3.0.CO;2-4

[34] Brand M. The sites and topology of mitochondrial superoxide production. Exp Gerontol. 2010 8;45(7–8):466–72. doi: 10.1016/j.exger.2010.01.003 20064600 10.1016/j.exger.2010.01.003 PMC2879443

[35] Ristow M, Schmeisser S. Extending life span by increasing oxidative stress. Free Radic Biol Med. 2011;51(2):327–36. doi: 10.1016/j.freeradbiomed.2011.05.010 21619928 10.1016/j.freeradbiomed.2011.05.010

[36] Van Raamsdonk JM, Hekimi S. Superoxide dismutase is dispensable for normal animal lifespan. Proc Natl Acad Sci U S A. 2012 4 10;109(15):5785–90. doi: 10.1073/pnas.1116158109 22451939 10.1073/pnas.1116158109 PMC3326508

[37] Bansal A, Zhu LJ, Yen K, Tissenbaum H a. Uncoupling lifespan and healthspan in Caenorhabditis elegans longevity mutants. Proc Natl Acad Sci. 2015;112(3):E277–86. doi: 10.1073/pnas.1412192112 25561524 10.1073/pnas.1412192112 PMC4311797

[38] Yee C, Yang W, Hekimi S. The intrinsic apoptosis pathway mediates the pro-longevity response to mitochondrial ROS in C elegans. Cell. 2014 5;157(4):897–909. doi: 10.1016/j.cell.2014.02.055 24813612 10.1016/j.cell.2014.02.055 PMC4454526

[39] Munkácsy E, Rea SL. The paradox of mitochondrial dysfunction and extended longevity. Exp Gerontol. 2014;56:221–33. doi: 10.1016/j.exger.2014.03.016 24699406 10.1016/j.exger.2014.03.016 PMC4104296

[40] Lee SJ, Hwang AB, Kenyon C. Inhibition of respiration extends C. elegans life span via reactive oxygen species that increase HIF-1 activity. Curr Biol. 2010;20(23):2131–6. doi: 10.1016/j.cub.2010.10.057 21093262 10.1016/j.cub.2010.10.057 PMC3058811

[41] Back P, Braeckman BP, Matthijssens F. ROS in aging Caenorhabditis elegans: damage or signaling? Oxid Med Cell Longev. 2012 1;2012:608478 doi: 10.1155/2012/608478 22966416 10.1155/2012/608478 PMC3431105

[42] An JH, Blackwell TK. SKN-1 links C. elegans mesendodermal specification to a conserved oxidative stress response. Genes Dev. 2003 8 1;17(15):1882–93. doi: 10.1101/gad.1107803 12869585 10.1101/gad.1107803 PMC196237

[43] Tullet JM a, Hertweck M, An JH, Baker J, Hwang JY, Liu S, et al Direct inhibition of the longevity-promoting factor SKN-1 by insulin-like signaling in C. elegans. Cell. 2008 3 21;132(6):1025–38. doi: 10.1016/j.cell.2008.01.030 18358814 10.1016/j.cell.2008.01.030 PMC2367249

[44] Oliveira RP, Abate JP, Dilks K, Landis J, Ashraf J, Murphy CT, et al Condition-adapted stress and longevity gene regulation by Caenorhabditis elegans SKN-1/Nrf. Aging Cell. 2009;8(5):524–41. doi: 10.1111/j.1474-9726.2009.00501.x 19575768 10.1111/j.1474-9726.2009.00501.x PMC2776707

[45] Shen C, Nettleton D, Jiang M, Kim SK, Powell-Coffman JA. Roles of the HIF-1 hypoxia-inducible factor during hypoxia response in Caenorhabditis elegans. J Biol Chem. 2005;280(21):20580–8. doi: 10.1074/jbc.M501894200 15781453 10.1074/jbc.M501894200

[46] Baruah A, Chang H, Hall M, Yuan J, Gordon S, Johnson E, et al CEP-1, the Caenorhabditis elegans p53 Homolog, Mediates Opposing Longevity Outcomes in Mitochondrial Electron Transport Chain Mutants. PLoS Genet. 2014;10(2). 10.1371/journal.pgen.1004097 PMC3937132 24586177

[47] Miyako K, Kai Y, Irie T, Takeshige K, Kang D. The content of intracellular mitochondrial DNA is decreased by 1- methyl-4-phenylpyridinium ion (MPP+). J Biol Chem. 1997;272(15):9605–8. 9092484 10.1074/jbc.272.15.9605

[48] Lee H-C, Wei Y-H. Mitochondrial biogenesis and mitochondrial DNA maintenance of mammalian cells under oxidative stress. Int J Biochem Cell Biol. 2005 4;37(4):822–34. doi: 10.1016/j.biocel.2004.09.010 15694841 10.1016/j.biocel.2004.09.010

[49] Murphy MP. How mitochondria produce reactive oxygen species. Biochem J. 2009 1 1;417(1):1–13. doi: 10.1042/BJ20081386 19061483 10.1042/BJ20081386 PMC2605959

[50] Jiang B, Hebert VY, Li Y, Mathis JM, Alexander JS, Dugas TR. HIV antiretroviral drug combination induces endothelial mitochondrial dysfunction and reactive oxygen species production, but not apoptosis. Toxicol Appl Pharmacol. 2007 10 1;224(1):60–71. doi: 10.1016/j.taap.2007.06.010 17669453 10.1016/j.taap.2007.06.010

[51] Ballinger SW, Patterson C, Yan CN, Doan R, Burow DL, Young CG, et al Hydrogen peroxide- and peroxynitrite-induced mitochondrial DNA damage and dysfunction in vascular endothelial and smooth muscle cells. Circ Res. 2000;86(9):960–6. 10807868 10.1161/01.res.86.9.960

[52] Yan LJ, Sohal RS. Mitochondrial adenine nucleotide translocase is modified oxidatively during aging. Proc Natl Acad Sci U S A. 1998;95(22):12896–901. 9789011 10.1073/pnas.95.22.12896 PMC23645

[53] Cochemé HM, Murphy MP. Complex I is the major site of mitochondrial superoxide production by paraquat. J Biol Chem. 2008;283(4):1786–98. doi: 10.1074/jbc.M708597200 18039652 10.1074/jbc.M708597200

[54] Palmeira CM, Moreno AJ, Madeira VMC. Mitochondrial bioenergetics is affected by the herbicide paraquat. Biochim Biophys Acta—Bioenerg. 1995;1229(2):187–92. 10.1016/0005-2728(94)00202-g 7727498

[55] Hamanaka RB, Chandel NS. Mitochondrial reactive oxygen species regulate cellular signaling and dictate biological outcomes. Trends Biochem Sci. 2010;35(9):505–13. doi: 10.1016/j.tibs.2010.04.002 20430626 10.1016/j.tibs.2010.04.002 PMC2933303

[56] Varghese F, Atcheson E, Bridges HR, Hirst J. Characterization of clinically-identified mutations in NDUFV1, the flavin-binding subunit of respiratory complex I, using a yeast model system. Hum Mol Genet. 2015;1–29. 10.1093/hmg/ddv344 PMC4614703 26345448

[57] Sivitz WI, Yorek M a. Mitochondrial dysfunction in diabetes: from molecular mechanisms to functional significance and therapeutic opportunities. Antioxid Redox Signal. 2010;12(4):537–77. doi: 10.1089/ars.2009.2531 19650713 10.1089/ars.2009.2531 PMC2824521

[58] Pulliam DA, Bhattacharya A, Van Remmen H. Mitochondrial Dysfunction in Aging and Longevity: A Causal or Protective Role? Antioxid Redox Signal. 2013;19(12):1373–87. doi: 10.1089/ars.2012.4950 23025472 10.1089/ars.2012.4950

[59] Cristina D, Cary M, Lunceford A, Clarke C, Kenyon C. A regulated response to impaired respiration slows behavioral rates and increases lifespan in Caenorhabditis elegans. PLoS Genet. 2009 4;5(4):e1000450 doi: 10.1371/journal.pgen.1000450 19360127 10.1371/journal.pgen.1000450 PMC2660839

[60] Ames BN. Optimal micronutrients delay mitochondrial decay and age-associated diseases. Mech Ageing Dev. 2010;131(7–8):473–9. doi: 10.1016/j.mad.2010.04.005 20420847 10.1016/j.mad.2010.04.005

[61] Tarnopolsky M a. The mitochondrial cocktail: rationale for combined nutraceutical therapy in mitochondrial cytopathies. Adv Drug Deliv Rev. 2008;60(13–14):1561–7. doi: 10.1016/j.addr.2008.05.001 18647623 10.1016/j.addr.2008.05.001

[62] Allard JP, Aghdassi E, Chau J, Tam C, Kovacs CM, Salit IE, et al Effects of vitamin E and C supplementation on oxidative stress and viral load in HIV-infected subjects. AIDS. 1998 9 10;12(13):1653–9. 9764785 10.1097/00002030-199813000-00013

[63] Mondal D, Pradhan L, Ali M, Agrawal KC. HAART drugs induce oxidative stress in human endothelial cells and increase endothelial recruitment of mononuclear cells: exacerbation by inflammatory cytokines and amelioration by antioxidants. Cardiovasc Toxicol. 2004 1;4(3):287–302. 15470276 10.1385/ct:4:3:287

[64] de la Asunción JG, Del Olmo ML, Gómez-Cambronero LG, Sastre J, Pallardó F V, Viña J. AZT induces oxidative damage to cardiac mitochondria: protective effect of vitamins C and E. Life Sci. 2004 11 19;76(1):47–56. doi: 10.1016/j.lfs.2004.06.020 15501479 10.1016/j.lfs.2004.06.020

[65] Lopez O, Bonnefont-Rousselot D, Edeas M, Emerit J, Bricaire F. Could antioxidant supplementation reduce antiretroviral therapy-induced chronic stable hyperlactatemia? Biomed Pharmacother. 2003 5;57(3–4):113–6. 12818471 10.1016/s0753-3322(03)00017-9

[66] Mehta S, Fawzi W. Effects of vitamins, including vitamin A, on HIV/AIDS patients. Vitam Horm. 2007 1;75:355–83. doi: 10.1016/S0083-6729(06)75013-0 17368322 10.1016/S0083-6729(06)75013-0

[67] Patrick L. Nutrients and HIV: part one—beta carotene and selenium. Altern Med Rev. 1999 12;4(6):403–13. 10608913

[68] Patrick L. Nutrients and HIV: part two—vitamins A and E, zinc, B-vitamins, and magnesium. Altern Med Rev. 2000 2;5(1):39–51. 10696118

[69] Patrick L. Nutrients and HIV: part three—N-acetylcysteine, alpha-lipoic acid, L-glutamine, and L-carnitine. Altern Med Rev. 2000 8;5(4):290–305. 10956377

[70] Milazzo L, Menzaghi B, Caramma I, Nasi M, Sangaletti O, Cesari M, et al Effect of antioxidants on mitochondrial function in HIV-1-related lipoatrophy: a pilot study. AIDS Res Hum Retroviruses. 2010 11;26(11):1207–14. doi: 10.1089/aid.2010.0024 20977356 10.1089/aid.2010.0024

[71] Dröge W, Breitkreutz R. N-acetyl-cysteine in the therapy of HIV-positive patients. Curr Opin Clin Nutr Metab Care. 1999 11;2(6):493–8. 10678679 10.1097/00075197-199911000-00011

[72] Luz AL, Meyer JN. Effects of reduced mitochondrial DNA content on secondary mitochondrial toxicant exposure in Caenorhabditis elegans. Mitochondrion. 2016 8 23;30:2–11. 10.1016/j.mito.2016.08.014 PMC5023498 27566481

[73] Wang X, Chai H, Lin PH, Yao Q, Chen C. Roles and mechanisms of human immunodeficiency virus protease inhibitor ritonavir and other anti-human immunodeficiency virus drugs in endothelial dysfunction of porcine pulmonary arteries and human pulmonary artery endothelial cells. Am J Pathol. 2009 3;174(3):771–81. doi: 10.2353/ajpath.2009.080157 19218343 10.2353/ajpath.2009.080157 PMC2665739

[74] Sun R, Eriksson S, Wang L. Zidovudine induces downregulation of mitochondrial deoxynucleoside kinases: implications for mitochondrial toxicity of antiviral nucleoside analogs. Antimicrob Agents Chemother. 2014 11 1;58(11):6758–66. doi: 10.1128/AAC.03613-14 25182642 10.1128/AAC.03613-14 PMC4249380

[75] Hukezalie KR, Thumati NR, Côté HCF, Wong JMY. In Vitro and Ex Vivo Inhibition of Human Telomerase by Anti-HIV Nucleoside Reverse Transcriptase Inhibitors (NRTIs) but Not by Non-NRTIs. PLoS One. 2012 1;7(11):e47505 doi: 10.1371/journal.pone.0047505 23166583 10.1371/journal.pone.0047505 PMC3499584

[76] O’Rourke EJ, Soukas AA, Carr CE, Ruvkun G. C. elegans major fats are stored in vesicles distinct from lysosome-related organelles. Cell Metab. 2009 11;10(5):430–5. doi: 10.1016/j.cmet.2009.10.002 19883620 10.1016/j.cmet.2009.10.002 PMC2921818

[77] Mitchell DH, Stiles JW, Santelli J, Sanadi DR. Synchronous growth and aging of Caenorhabditis elegans in the presence of fluorodeoxyuridine. J Gerontol. 1979 1;34(1):28–36. 153363 10.1093/geronj/34.1.28

[78] Burns AR, Wallace IM, Wildenhain J, Tyers M, Giaever G, Bader GD, et al A predictive model for drug bioaccumulation and bioactivity in Caenorhabditis elegans. Nat Chem Biol. 2010;6(7):549–57. doi: 10.1038/nchembio.380 20512140 10.1038/nchembio.380

[79] Holden-Dye L, Walker R. Anthelmintic drugs [Internet]. WormBook. 2007 [cited 2015 Sep 21]. http://www.wormbook.org/chapters/www_anthelminticdrugs/anthelminticdrugs.html#sec3 10.1895/wormbook.1.143.1 PMC4781348 17988075

[80] Munch-Petersen B, Cloos L, Tyrsted G, Eriksson S. Diverging substrate specificity of pure human thymidine kinases 1 and 2 against antiviral dideoxynucleosides. J Biol Chem. 1991 5 15;266(14):9032–8. 2026611

[81] Wang L, Sun R, Eriksson S. The kinetic effects on thymidine kinase 2 by enzyme-bound dTTP may explain the mitochondrial side effects of antiviral thymidine analogs. Antimicrob Agents Chemother. 2011 6;55(6):2552–8. doi: 10.1128/AAC.00109-11 21444706 10.1128/AAC.00109-11 PMC3101437

[82] Anderson PL, Kakuda TN, Lichtenstein K a. The cellular pharmacology of nucleoside- and nucleotide-analogue reverse-transcriptase inhibitors and its relationship to clinical toxicities. Clin Infect Dis. 2004 3 1;38(5):743–53. doi: 10.1086/381678 14986261 10.1086/381678

[83] Hasegawa K, Miwa S, Tajima T, Tsutsumiuchi K, Taniguchi H, Miwa J. A rapid and inexpensive method to screen for common foods that reduce the action of acrylamide, a harmful substance in food. Toxicol Lett. 2007 12 10;175(1–3):82–8. doi: 10.1016/j.toxlet.2007.09.013 18023302 10.1016/j.toxlet.2007.09.013

[84] Bolger AM, Lohse M, Usadel B. Trimmomatic: A flexible trimmer for Illumina sequence data. Bioinformatics. 2014;30(15):2114–20. doi: 10.1093/bioinformatics/btu170 24695404 10.1093/bioinformatics/btu170 PMC4103590

[85] Kim D, Pertea G, Trapnell C, Pimentel H, Kelley R, Salzberg SL. TopHat2: accurate alignment of transcriptomes in the presence of insertions, deletions and gene fusions. Genome Biol. 2013;14(4):R36 doi: 10.1186/gb-2013-14-4-r36 23618408 10.1186/gb-2013-14-4-r36 PMC4053844

[86] Langmead B, Salzberg SL. Fast gapped-read alignment with Bowtie 2. Vol. 9, Nature Methods. 2012 p. 357–9. 10.1038/nmeth.1923 PMC3322381 22388286

[87] Anders S, Pyl PT, Huber W. HTSeq A Python framework to work with high-throughput sequencing data. bioRxiv. 2014. 10.1093/bioinformatics/btu638 PMC4287950 25260700

[88] Anders S, Huber W. Differential expression analysis for sequence count data. Genome Biol. 2010;11(10):R106 doi: 10.1186/gb-2010-11-10-r106 20979621 10.1186/gb-2010-11-10-r106 PMC3218662

[89] Yang W, Hekimi S. Two modes of mitochondrial dysfunction lead independently to lifespan extension in Caenorhabditis elegans. Aging Cell. 2010 6;9(3):433–47. doi: 10.1111/j.1474-9726.2010.00571.x 20346072 10.1111/j.1474-9726.2010.00571.x

[90] Yang W, Li J, Hekimi S. A Measurable increase in oxidative damage due to reduction in superoxide detoxification fails to shorten the life span of long-lived mitochondrial mutants of Caenorhabditis elegans. Genetics. 2007 12;177(4):2063–74. doi: 10.1534/genetics.107.080788 18073424 10.1534/genetics.107.080788 PMC2219504

[91] Lagido C, Pettitt J, Flett A, Glover LA. Bridging the phenotypic gap: real-time assessment of mitochondrial function and metabolism of the nematode Caenorhabditis elegans. BMC Physiol. 2008 1;8:7 doi: 10.1186/1472-6793-8-7 18384668 10.1186/1472-6793-8-7 PMC2364618

[92] Lawless J. Statistical Models and Methods for Lifetime Data. 2nd ed New York: John Wiley & Sons, 2011; 1982.

[93] Nazir A, Sammi SR, Singh P, Tripathi RK. Trans-cellular introduction of HIV-1 protein Nef induces pathogenic response in Caenorhabditis elegans. PLoS One. 2010 1;5(12):e15312 doi: 10.1371/journal.pone.0015312 21179446 10.1371/journal.pone.0015312 PMC3001482

[94] Mattson MP. Hormesis defined. Ageing Res Rev. 2008;7(1):1–7. doi: 10.1016/j.arr.2007.08.007 18162444 10.1016/j.arr.2007.08.007 PMC2248601

[95] Schulz TJ, Zarse K, Voigt A, Urban N, Birringer M, Ristow M. Glucose Restriction Extends Caenorhabditis elegans Life Span by Inducing Mitochondrial Respiration and Increasing Oxidative Stress. Cell Metab. 2007;6(4):280–93. doi: 10.1016/j.cmet.2007.08.011 17908557 10.1016/j.cmet.2007.08.011

