# Supplementary figures and images for: Beyond the polymerase-γ theory: Production of ROS as a mode of NRTI-induced mitochondrial toxicity
Source: PLoS One. 2017 Nov 2;12(11):e0187424. doi: 10.1371/journal.pone.0187424 (PMC5667870; doi:10.1371/journal.pone.0187424)

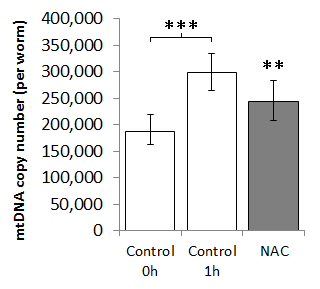

Supplement: S1 Fig — NAC reduced the normal mtDNA copy number increase during 1h exposure. 100μM NAC decrease mtDNA copy number compared to control nematodes after 1h exposure (dark grey vs 1h control). mtDNA copy number increases during nematode development and therefore rises (control 0h vs control 1h). Error bars show the 95% C.I. (df = 51). Significance was determined using a two-tailed student’s T-test assuming unequal variances. ** = P-value <0.01, *** = P-value <0.001 compared to control animals at 1h, and Control 1h vs Control 0h. (TIF) [file pone.0187424.s001.tif]

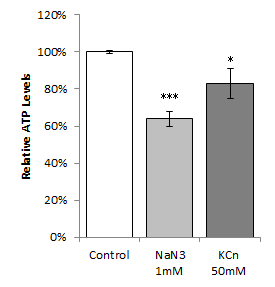

Supplement: S2 Fig — Complex IV inhibitors, sodium azide (NaN3) and potassium cyanide (KCn), reduced ATP production. 1mM NaN3 and 50mM KCn reduced in vivo ATP levels measured after 2 minutes of exposure. Statistics were calculated with a two-way ANOVA with replication, compared to the control. * = P<0.05, *** = P<0.001. (TIF) [file pone.0187424.s002.tif]

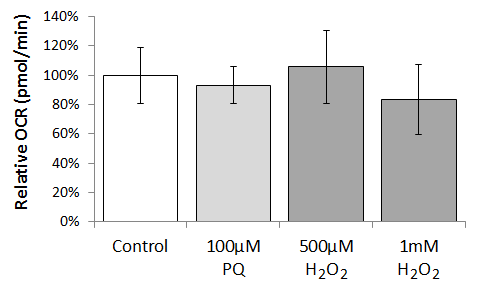

Supplement: S3 Fig — Immediate OCR rates after injection of H2O2 and paraquat (PQ) do not change. The lack of these pro-oxidants to have an immediate effect on OCR supports the observations that they do not directly inhibit MRC complex function at the concentrations used in this study [53,54]. (TIF) [file pone.0187424.s003.tif]

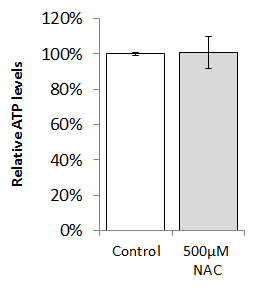

Supplement: S4 Fig — ATP levels did not change upon exposure to 500μM NAC. In vivo ATP levels measured after 2 minutes of exposure. (TIF) [file pone.0187424.s004.tif]

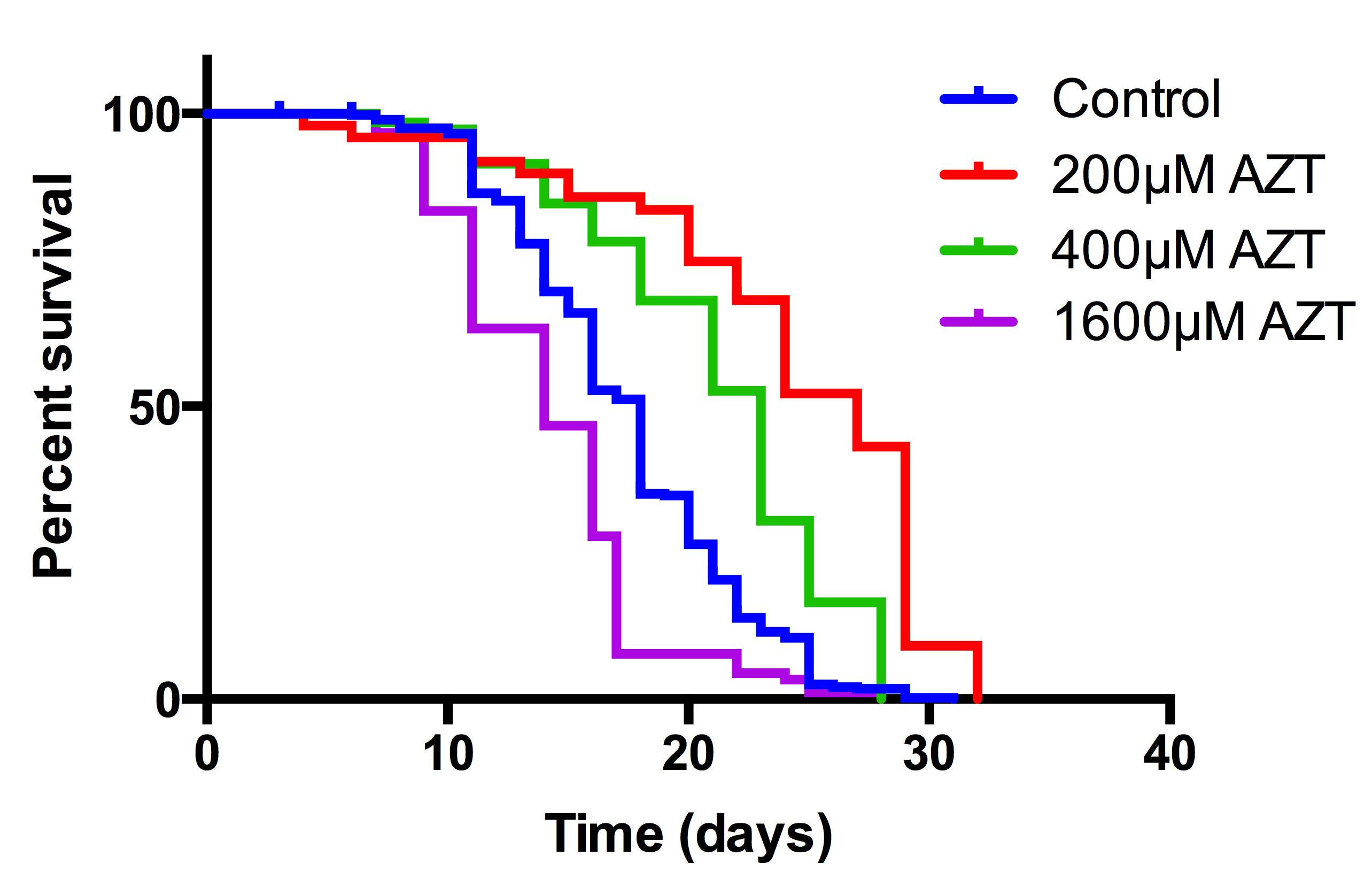

Supplement: S5 Fig — In accordance with mitohormetic events triggered by inhibited MRC function and an increase in ROS, 200μM AZT caused significant lifespan extension in C. elegans. An increase in AZT concentration above 200μM steadily decreased life span in L4 animals, indicating that NRTIs cause a biphasic dose response, reconfirming hormesis [94]. Increasing the concentration of AZT above a threshold of approximately 200μM reversed the life extension seen with 200μM. Animals were exposed from the L4 larval stage at 20°C. Asterisks indicate significance as calculated with the Mantel-Cox test compared to the control: * = P<0.05, ** = P<0.01, *** = P<0.001, n.s. = not significant. (TIF) [file pone.0187424.s005.tif]

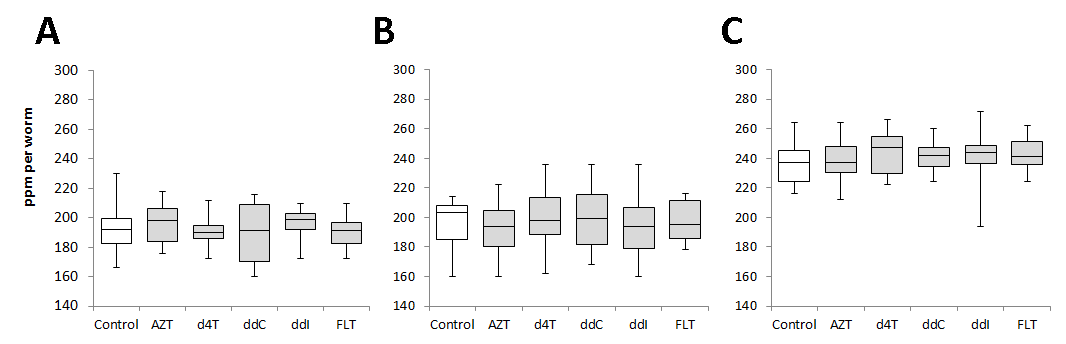

Supplement: S6 Fig — In C. elegans, caloric restriction during development or adulthood can cause lifespan extension by increasing mitochondrial activity and prompting the expression of genes involved in stress resistance and longevity [95]. Although pharyngeal pumping is not a direct measure of feeding we tested if the changes in lifespan traits we observed were caused by caloric restriction due to a reduced food intake (reduced pharyngeal pumping). We exposed nematodes from the L4 larval stage and counted pharyngeal pumping rates at 2, 6, and 24h. No significant change in pumping rate was seen at time points 2, 6, and 24h. Taken together, these results suggest that the observed extension in lifespan is likely not caused by caloric restriction. A: 2h exposure, B: 6h exposure, and C: 24h exposure to NRTIs does not alter pharyngeal pumping rates per minute (ppm) per worm. NRTI concentration = 200μM. L4 animals were exposed to NRTIs and pharyngeal pumping rates of 10 animals from each condition were manually counted under a Leica M80 stereo microscope for the duration of 30 seconds. To assess short-term and long-term effects, drug exposure times were 2, 6, 24, and 48h. Dead and non-pumping animals were not included. 1 pump was defined as 1 grinder movement. Pumping rates were measured at room temperature (approximately 22°C) on NGM agar with confluent lawns of OP50 bacteria. (TIF) [file pone.0187424.s006.tif]

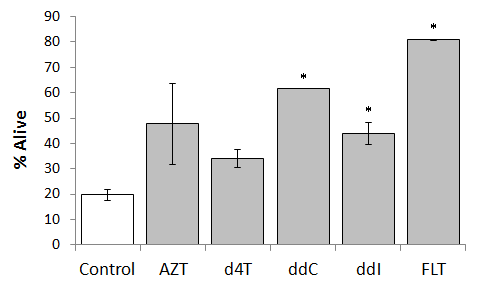

Supplement: S7 Fig — 4mM paraquat (PQ) survival assay of NRTI exposed animals. Statistics were calculated by two sided student’s t-test assuming unequal variance, compared to the control of that same time point (72h of PQ exposure). (TIF) [file pone.0187424.s007.tif]
