# Supplementary material for: Beyond the polymerase-γ theory: Production of ROS as a mode of NRTI-induced mitochondrial toxicity
Source: PLoS One. 2017 Nov 2;12(11):e0187424. doi: 10.1371/journal.pone.0187424 (PMC5667870; doi:10.1371/journal.pone.0187424)
Supplement: S1 Table — The number of sigmoidal body bends per worm per minute can be used as a proxy for fitness. Anti-oxidant concentration = 100μM. Statistics were calculated by two sided student’s t-test assuming unequal variance compared to control of that same time point. n.s. = not significant. (DOCX) [file pone.0187424.s008.docx]

|  | Control | NAC |
| --- | --- | --- |
| 24h | 129.4 (±13.7) | 127.1 (±19.5) n.s. |
| 48h | 119.5 (±17) | 118.7 (±9.1) n.s. |
| 72h | 107.7 (±16.3) | 101.3 (±20.8) n.s. |
